# Supplementary material for: Prevalence and social determinants of anxiety and depressive disorders and symptoms among adults in Ghana: A systematic review and meta-analysis
Source: Glob Ment Health (Camb). 2025 Dec 19;13:e5. doi: 10.1017/gmh.2025.10122 (PMC12835950; doi:10.1017/gmh.2025.10122)
Supplement: Awortwe et al. supplementary material [file S2054425125101222sup001.docx]

**Prevalence and social determinants of anxiety and depressive disorders and symptoms among adults in Ghana: a systematic review and meta-analysis**

**Supplementary materials**

Contents

[Appendix A. PRISMA 2020 checklist 2](#_Toc205930319)

[Appendix B. Changes to protocol 7](#_Toc205930320)

[Appendix C: PubMed search strategy and overview of nine electronic databases 8](#_Toc205930321)

[Appendix D: Joanna Briggs Institute (JBI) critical appraisal checklist for prevalence studies and rationale for response options 11](#_Toc205930322)

[Appendix E. Quality assessment of included studies 15](#_Toc205930323)

[Appendix F. References of included studies 19](#_Toc205930324)

[Appendix G. All reasons for exclusion (N= 234) 23](#_Toc205930325)

[Appendix H. References to excluded studies 46](#_Toc205930326)

[Appendix I. Funnel plot for publication bias for anxiety disorders and symptoms 65](#_Toc205930327)

[Appendix J. Funnel plot for publication bias for depressive disorders and symptoms 66](#_Toc205930328)

[Appendix K. Forest and funnel plot for publication bias for symptoms of psychological distress 67](#_Toc205930329)

[Appendix L. Subgroup analyses of prevalence of anxiety disorders and symptoms (n=19) 68](#_Toc205930330)

[Appendix M. Regional distribution of prevalence of anxiety disorders and symptoms 70](#_Toc205930331)

[Appendix N. Subgroup analyses of prevalence of depressive disorders and symptoms (n=30) 71](#_Toc205930332)

[Appendix O. Regional distribution of prevalence of depressive disorders and symptoms 73](#_Toc205930333)

# Appendix A. PRISMA 2020 checklist

| Section and Topic | Item # | Checklist item | Location where item is reported |
| --- | --- | --- | --- |
| TITLE | | |  |
| Title | 1 | Identify the report as a systematic review. | Review protocol (p. 1)  Review results (p. 1) |
| ABSTRACT | | |  |
| Abstract | 2 | See the PRISMA 2020 for Abstracts checklist. | Review results (p. 1) |
| INTRODUCTION | | |  |
| Rationale | 3 | Describe the rationale for the review in the context of existing knowledge. | Review protocol (p. 1-2)  Review results (p. 3-5) |
| Objectives | 4 | Provide an explicit statement of the objective(s) or question(s) the review addresses. | Review protocol (p. 2)  Review results (p. 5) |
| METHODS | | |  |
| Eligibility criteria | 5 | Specify the inclusion and exclusion criteria for the review and how studies were grouped for the syntheses. | Review protocol (p. 3)  Review results (p. 6-7) |
| Information sources | 6 | Specify all databases, registers, websites, organisations, reference lists and other sources searched or consulted to identify studies. Specify the date when each source was last searched or consulted. | Review protocol (p. 3-4)  Review results (p. 5) |
| Search strategy | 7 | Present the full search strategies for all databases, registers and websites, including any filters and limits used. | Review protocol (Appendix 3)  Review results  (p. 5 & Appendix C) |
| Selection process | 8 | Specify the methods used to decide whether a study met the inclusion criteria of the review, including how many reviewers screened each record and each report retrieved, whether they worked independently, and if applicable, details of automation tools used in the process. | Review protocol (p. 4)  Review results (p. 7) |
| Data collection process | 9 | Specify the methods used to collect data from reports, including how many reviewers collected data from each report, whether they worked independently, any processes for obtaining or confirming data from study investigators, and if applicable, details of automation tools used in the process. | Review protocol (p. 4)  Review results (p. 5, 7) |
| Data items | 10a | List and define all outcomes for which data were sought. Specify whether all results that were compatible with each outcome domain in each study were sought (e.g. for all measures, time points, analyses), and if not, the methods used to decide which results to collect. | Review protocol (p. 4)  Review results (p. 7) |
|  | 10b | List and define all other variables for which data were sought (e.g. participant and intervention characteristics, funding sources). Describe any assumptions made about any missing or unclear information. | Review protocol (p. 4)  Review protocol (p. 7)  Review results (p. 7) |
| Study risk of bias assessment | 11 | Specify the methods used to assess risk of bias in the included studies, including details of the tool(s) used, how many reviewers assessed each study and whether they worked independently, and if applicable, details of automation tools used in the process. | Review protocol (p. 4)  Review results (p. 8) |
| Effect measures | 12 | Specify for each outcome the effect measure(s) (e.g. risk ratio, mean difference) used in the synthesis or presentation of results. | Review protocol (p. 4)  Review results (p. 8) |
| Synthesis methods | 13a | Describe the processes used to decide which studies were eligible for each synthesis (e.g. tabulating the study intervention characteristics and comparing against the planned groups for each synthesis (item #5)). | Review results (p. 6-9) |
|  | 13b | Describe any methods required to prepare the data for presentation or synthesis, such as handling of missing summary statistics, or data conversions. | Review protocol (p. 4-5) Review results (p.8- 9) |
|  | 13c | Describe any methods used to tabulate or visually display results of individual studies and syntheses. | Review results (p. 8-9) |
|  | 13d | Describe any methods used to synthesize results and provide a rationale for the choice(s). If meta-analysis was performed, describe the model(s), method(s) to identify the presence and extent of statistical heterogeneity, and software package(s) used. | Review protocol (p. 4-5)  Review results (p. 8-9) |
|  | 13e | Describe any methods used to explore possible causes of heterogeneity among study results (e.g. subgroup analysis, meta-regression). | Review protocol (p. 5) Review results (p. 8-9) |
|  | 13f | Describe any sensitivity analyses conducted to assess robustness of the synthesized results. | Review protocol (p. 5) Review results (p. 8) |
| Reporting bias assessment | 14 | Describe any methods used to assess risk of bias due to missing results in a synthesis (arising from reporting biases). | Review protocol (p. 4) Review results (p.8) |
| Certainty assessment | 15 | Describe any methods used to assess certainty (or confidence) in the body of evidence for an outcome. | N/A |
| **RESULTS** | | |  |
| Study selection | 16a | Describe the results of the search and selection process, from the number of records identified in the search to the number of studies included in the review, ideally using a flow diagram. | Review results (p. 10) |
|  | 16b | Cite studies that might appear to meet the inclusion criteria, but which were excluded, and explain why they were excluded. | Review results (Appendices G and H) |
| Study characteristics | 17 | Cite each included study and present its characteristics. | Review results (p. 25-37; Appendix F) |
| Risk of bias in studies | 18 | Present assessments of risk of bias for each included study. | Review results (p. 11 and Appendix D & E) |
| Results of individual studies | 19 | For all outcomes, present, for each study: (a) summary statistics for each group (where appropriate) and (b) an effect estimates and its precision (e.g. confidence/credible interval), ideally using structured tables or plots. | Review results (p. 10) |
| Results of syntheses | 20a | For each synthesis, briefly summarise the characteristics and risk of bias among contributing studies. | Review results (p. 10-11) |
|  | 20b | Present results of all statistical syntheses conducted. If meta-analysis was done, present for each the summary estimate and its precision (e.g. confidence/credible interval) and measures of statistical heterogeneity. If comparing groups, describe the direction of the effect. | Review results (p. 11-13) |
|  | 20c | Present results of all investigations of possible causes of heterogeneity among study results. | Review results (p. 13-14) |
|  | 20d | Present results of all sensitivity analyses conducted to assess the robustness of the synthesized results. | Review results (p. 11) |
| Reporting biases | 21 | Present assessments of risk of bias due to missing results (arising from reporting biases) for each synthesis assessed. | Review results (p. 10-17) |
| Certainty of evidence | 22 | Present assessments of certainty (or confidence) in the body of evidence for each outcome assessed. | N/A |
| **DISCUSSION** | | |  |
| Discussion | 23a | Provide a general interpretation of the results in the context of other evidence. | Review results (p. 18-21) |
|  | 23b | Discuss any limitations of the evidence included in the review. | Review results (p. 21) |
|  | 23c | Discuss any limitations of the review processes used. | Review results (p. 21) |
|  | 23d | Discuss implications of the results for practice, policy, and future research. | Review results (p. 21-22) |
| **OTHER INFORMATION** | | |  |
| Registration and protocol | 24a | Provide registration information for the review, including register name and registration number, or state that the review was not registered. | Review protocol (p. 1)  Review results (p. 5) |
|  | 24b | Indicate where the review protocol can be accessed, or state that a protocol was not prepared. | Review results (p. 5) |
|  | 24c | Describe and explain any amendments to information provided at registration or in the protocol. | Review results (Appendix B) |
| Support | 25 | Describe sources of financial or non-financial support for the review, and the role of the funders or sponsors in the review. | Review protocol (p. 6)  Review results (p. 24) |
| Competing interests | 26 | Declare any competing interests of review authors. | Review protocol (p. 6)  Review results (p. 24) |
| Availability of data, code and other materials | 27 | Report which of the following are publicly available and where they can be found: template data collection forms; data extracted from included studies; data used for all analyses; analytic code; any other materials used in the review. | Review results (p. 18) |

# **Appendix B. Changes to protocol**

Prior to analysis, the following changes were made to the published protocol (Awortwe *et al.* 2024), with protocol amendments recorded in PROSPERO:

- In the original protocol, our aims were to (1) examine the prevalence of anxiety and depression among adults in Ghana and (2) explore social determinants potentially associated with anxiety and depression. Given we include both studies that estimate the prevalence of a clinical anxiety and depressive disorders as well as the prevalence of anxiety or depressive symptoms, we have slightly revised the aims to emphasise this distinction.
- We have clarified that examining the prevalence of symptoms of psychological distress is a secondary aim of the review.
- Initially, we planned to conduct a subgroup analysis to examine the moderating effect of sample size (<100 vs. ≥100) on prevalence estimates. However, as per the JBI critical appraisal for prevalence studies, a sample size of ≥384 relative to ≥100 is considered adequate. Therefore, sample size (<384 vs. ≥384) was used for the subgroup analysis.
- We conducted a subgroup analysis to examine the moderating effect of region (s) and population type on the prevalence of anxiety and depressive disorders and symptoms. This was necessary to examine the effect of other sources of heterogeneity on prevalence estimates.
- We planned to perform metaregression, as an extension of subgroup analysis to examine the effect of publication year (continuous moderator) on the pooled prevalence of anxiety and depressive disorders and symptoms. Similar choice has been made in other systematic reviews (Cai *et al.* 2024; Nisar *et al.* 2020). However, we decided to not to proceed with this analysis as publication year was found not to be representative of the timing of data collection, which is more relevant to understanding changes in prevalence over time.

# **Appendix C: PubMed search strategy and overview of nine electronic databases**

| **#** | **Searches** | **Comment** |
| --- | --- | --- |
| 1 | Mental health [Title/Abstract] OR  Mental disorders [Title/Abstract] OR  Mental disorder [Title/Abstract] OR  Mental illness [Title/Abstract] OR  Mental illnesses [Title/Abstract] OR  Anxiety [Title/Abstract] OR  Anxious [Title/Abstract] OR  Depress*[Title/Abstract] OR  Dysthymia [Title/Abstract] OR  Melancholy [Title/Abstract] OR  Mood [Title/Abstract] OR  Affective disorder [Title/Abstract] OR  Affective symptoms [Title/Abstract] OR  Negative affect [Title/Abstract] OR  distress [Title/Abstract] OR  Emotional stress [Title/Abstract] OR |  |
| 2 | Mental health [MeSH] OR  Mental disorders [MeSH] OR  Anxiety [MeSH] OR  Anxiety disorders [MeSH] OR  Depression [MeSH] OR  Mood disorders [MeSH] OR  Psychological Distress [MeSH] OR |  |
| 3 | 1 OR 2 |  |
| *Ghana* |  |  |
| *4* | Ghana [All fields] |  |
| *5* | Ghana [MeSH] |  |
| *6* | 4 OR 5 |  |
| *Final results* | 3 AND 6 |  |
| *Syntax* | (((((((((((((((((Mental health[Title/Abstract]) OR (Mental disorders[Title/Abstract])) OR (mental disorder[Title/Abstract])) OR (mental illness[Title/Abstract])) OR (mental illnesses[Title/Abstract])) OR (anxiety[Title/Abstract])) OR (anxious[Title/Abstract])) OR (depress*[Title/Abstract])) OR (dysthymia[Title/Abstract])) OR (melancholy[Title/Abstract])) OR (mood[Title/Abstract])) OR (affective disorder[Title/Abstract])) OR (affective symptoms[Title/Abstract])) OR (negative affect[Title/Abstract])) OR (distress[Title/Abstract])) OR (emotional stress[Title/Abstract])) OR ((((((((mental health[MeSH Terms]) OR (mental disorders[MeSH Terms])) OR (anxiety[MeSH Terms])) OR (anxiety disorders[MeSH Terms])) OR (depression[MeSH Terms])) OR (depressive disorder[MeSH Terms])) OR (mood disorders[MeSH Terms])) OR (psychological distress[MeSH Terms]))) AND ((Ghana) OR (Ghana[MeSH Terms])) | |
| **Nine electronic databases** | 1. African Index Medicus (AIM) 2. African Journals Online (AJOL) 3. Cumulative Index to Nursing and Allied Health Literature (CINAHL) 4. Excerpta Medica Database (Embase) 5. Ghana Medical Journal (GMJ) 6. Health Sciences Investigation (HIS) 7. MEDLINE (PubMed) 8. PsycINFO 9. SCOPUS | |

# **Appendix D: Joanna Briggs Institute (JBI) critical appraisal checklist for prevalence studies and rationale for response options**

| **JBI checklist items** | **Rationale for response options** | **Notes** |
| --- | --- | --- |
| Item I. Was the sample frame appropriate to address the target population? | **Yes:** The sampling frame was described and it included all members of the target population (e.g., a census, complete registry data, national database) and/or approximated the target population | See reference ^a^ below for JBI Guidance for full details |
|  | **No:** If the author did not clearly describe the target population, and/or a certain group was used (e.g., those working for an organisation, or one profession) but the results were inferred or generalised to the target population (i.e., working adults) |  |
|  | **Unclear:** If the author provided insufficient information about the target population and sampling frame, making it difficult to assess whether the sample frame appropriately represents the target population |  |
|  | **Not applicable:** If the study did not rely on a sample frame (e.g., total population studies, registry-based studies), or no sampling frame available was available for the adult population (e.g., adults with a rare disease), or the population of interest was hard to access or locate |  |
| Item 2: Were study participants recruited in an appropriate way? | **Yes:** If probability sampling method (e.g., random, stratified, cluster sampling) was used to select participants and the recruitment method is clearly described; or when everyone in the sampling frame will be included in the study |  |
|  | **No:** If non-probability sampling method (e.g., convenience, purposive) was used |  |
|  | **Unclear:** If the author did not clearly specify the sampling method or how participants were selected |  |
|  | **Not applicable:** If the study design did not require a structured sampling method (e.g., database studies where participants were not actively recruited) |  |
| Item 3: Was the sample size adequate? | **Yes:** If there was evidence that the authors conducted a sample size calculation to determine an adequate sample size. Sample size was considered adequate if the study was large enough (as in large national surveys) whereby a sample size calculation was not required, and/ or the sample size was ≥ 384 | See JBI Guidance for sample size calculation |
|  | **No:** If a sample size <384 was reported and there was no statistical justification for the chosen sample size |  |
|  | **Unclear:** If there was no statistical justification for the chosen sample size making it unclear if it was adequate for prevalence estimation, and/or there were discrepancies or inconsistencies in the description of the sample size |  |
| Item 4: Was the study population and setting clearly described? | **Yes:** If the study population (e.g., age, sex, eligibility criteria) and setting (e.g., location, type of institution, country, or region) were described in sufficient detail. We considered the description of the study population and setting sufficient if authors reported variables such as age, gender/sex, comorbidities, socioeconomic characteristics (e.g., educational background, and occupation), recruitment setting, and study location |  |
|  | **No:** If the study did not provide sufficient information about population demographics and study setting |  |
|  | **Unclear:** Some details about the study population and setting are provided but other important information was missing (e.g., age, and sex/gender) |  |
| Item 5: Was data analysis conducted with sufficient coverage of the identified sample? | **Yes:** If the study included all identified sample in the analysis, or exclusions were clearly justified; or response rate was reported for the entire sample and, where relevant for subgroups (e.g., based on age, sex/gender, health condition); or when authors presented characteristics of adult samples included and excluded from the final sample and there was not much difference between them |  |
|  | **No:** If a significant portion of the adult sample were excluded from the analysis without justification; or response rates were not reported, making it unclear whether certain subgroups of adults were underrepresented; or there was high response rate but the response rate for a certain subgroup (e.g., women) was quite low |  |
|  | **Unclear:** If the study did not provide enough detail to ascertain whether all eligible adults were included in the data analysis; or certain adult subgroups had lower response rates |  |
|  | **Not applicable:** If the study did not report data on subgroups and/ or the analysis did not require subgroup breakdowns (e.g., if no distinct subgroups existed); or the study included all adult sample) |  |
| Item 6: Were valid methods used for the identification of the condition?? | **Yes:** If there was evidence that anxiety and depressive disorders and symptoms, and symptoms of psychological distress were assessed based on valid assessment methods (e.g., diagnostic interview and validated self-report screening tools). Validity was determined based on evidence (construct validity, content validity, criterion validity, and reliability measures including internal consistency, test-retest reliability, and inter-rater reliability) provided in the study or validation paper(s) of assessment methods | All eligible studies were required to used valid assessment methods |
| Item 7: Was the condition measured in a standard, reliable way for all participants? | **Yes:** If there was information about the measurement procedures, expertise and/or training of data collectors, or evidence of measures taken to ensure that anxiety and depressive disorders and symptoms, and symptoms of psychological distress were measured in the same way for all participants |  |
|  | **No:** If the study failed to demonstrate that anxiety and depressive disorders and symptoms, and symptoms of psychological distress were measured in a standard and reliable way for all participants. This might occur if the data collectors were not adequately trained or educated on the measurement protocol, or if there was only one data collector without any mention of measures taken to ensure standard and reliability measurement of condition; or there were inconsistencies or variations in how the mental health conditions were assessed |  |
|  | **Unclear:** The study did not provide adequate information to establish how the measurement was conducted, and if it was measured in the same way for all participants |  |
| Item 8: Was there appropriate statistical analysis? | **Yes:** If the numerator and denominator were clearly reported, and percentages were given with confidence intervals and/or the methods section was detailed enough to identify the analytical technique used and how specific variables were measured. This response option was also applicable if analytical strategy aligned with assumptions associated with the strategy used |  |
|  | **No:** If prevalence was reported as percentages with no information on the numerator, denominator and/or confidence intervals. This response option was also applicable if the statistical technique did not align with the assumptions associated with the technique |  |
|  | **Unclear:** If the statistical method/ technique was not described in sufficient details and there was no motivation for the techniques used |  |
| Item 9: Was the response rate adequate, and if not, was low response bias managed appropriately? | **Yes:** If studies report response rate and/or clearly discuss or justify the response rate |  |
|  | **No:** If studies could have reported the response rate but they did not |  |
|  | **Unclear:** If the study does not report the response rate or did not provide enough information to ascertain whether low response rate was managed appropriately |  |

^a^ Munn Z, Moola S, Lisy K, Riitano D, Tufanaru C., 2015. Methodological guidance for systematic reviews of observational epidemiological studies reporting prevalence and incidence data. Int J Evid Based Healthc. 13(3):147–153.

# **Appendix E. Quality assessment of included studies**

| **NO.** | **Author** | **Appropriateness of sampling frame** | **Appropriateness of sample recruitment** | **Adequateness of sample size** | **Description of study subjects and setting in detail** | **Sufficiency of data analysis coverage** | **Validity of methods for identification of condition** | **Standard and reliable condition measurement** | **Appropriateness of statistical analysis** | **Adequateness of response rate a** | **Overall quality score** | **Overall quality rating** |
| --- | --- | --- | --- | --- | --- | --- | --- | --- | --- | --- | --- | --- |
| 1 | Canavan *et al.* 2013 | Yes | Yes | Yes | Yes | Unclear | Yes | Unclear | Yes | Yes | 7 | High |
| 2 | Kretchy *et al.* 2014 | Yes | Unclear | Yes | Yes | Yes | Yes | Unclear | Yes | Yes | 7 | High |
| 3 | Oppong Asante *et al.* 2015 | No | No | No | Yes | Unclear | Yes | Unclear | Yes | Unclear | 3 | Low |
| 4 | Ganu *et al.* 2018 | Yes | Unclear | No | Yes | Unclear | Yes | Unclear | Yes | Yes | 5 | Mod |
| 5 | Akpalu *et al.* 2018 | Yes | Yes | Yes | Yes | Unclear | Yes | Unclear | Yes | Unclear | 6 | Mod |
| 6 | Gyasi *et al.* 2019 | Yes | Yes | Yes | Unclear | Unclear | Yes | Yes | Yes | Unclear | 6 | Mod |
| 7 | Kugbey *et al.* 2018 | Yes | Yes | Yes | Yes | Yes | Yes | Unclear | Yes | Yes | 8 | High |
| 8 | Ademola *et al.* 2019 | Unclear | No | No | Yes | Unclear | Yes | Yes | Yes | Unclear | 4 | Mod |
| 9 | Awuah *et al.* 2019 | Yes | Yes | Yes | Yes | Unclear | Yes | Unclear | Yes | Unclear | 6 | Mod |
| 10 | Ofori-Atta *et al.* 2019 | Yes | Unclear | Yes | Yes | Yes | Yes | Unclear | Yes | Unclear | 6 | Mod |
| 11 | Nuvey *et al.* 2020 | Yes | Yes | No | Yes | No | Yes | Unclear | No | Unclear | 4 | Mod |
| 12 | **Adu et al.** 2021 | No | No | Yes | Yes | Unclear | Yes | Unclear | Yes | Unclear | 4 | Mod |
| 13 | Amoako *et al.* 2021 | Yes | No | No | Yes | Unclear | Yes | Yes | Yes | Unclear | 5 | Mod |
| 14 | Amu *et al.* 2021 | Yes | Yes | Yes | Yes | Yes | Yes | Yes | No | Unclear | 7 | High |
| 15 | Asare-Doku *et al.* 2021 | Yes | No | Yes | Yes | Unclear | Yes | Unclear | Yes | Yes | 6 | Mod |
| 16 | Boateng *et al.* 2021 | Unclear | No | Yes | Yes | Yes | Yes | Unclear | No | Unclear | 4 | Mod |
| 17 | Ofori et al., 2021 | No | No | Yes | Yes | Unclear | Yes | Unclear | Yes | Yes | 5 | Mod |
| 18 | Saah *et al.* 2021 | Yes | Yes | Yes | Yes | Yes | Yes | Yes | Yes | Unclear | 8 | High |
| 19 | Shaikh *et al.* 2021 | No | No | Yes | No | No | Yes | Unclear | Yes | Unclear | 3 | Low |
| 20 | Swaray *et al.* 2021 | Yes | Unclear | Yes | Yes | Unclear | Yes | Unclear | No | Unclear | 4 | Mod |
| 21 | Adjepong *et al.* 2022 | Yes | No | No | Yes | Unclear | Yes | Unclear | Yes | Yes | 5 | Mod |
| 22 | Arthur-Mensah *et al.* 2022 | Yes | Yes | No | Yes | Yes | Yes | Unclear | Yes | Yes | 7 | High |
| 23 | Boima *et al.* 2015 | Yes | No | Yes | Yes | Yes | Yes | Unclear | Yes | Unclear | 6 | Mod |
| 24 | Danquah and Mante 2022 | Yes | No | No | Yes | Unclear | Yes | Unclear | Yes | Unclear | 4 | Mod |
| 25 | Omuojine *et al.* 2022 | Yes | Unclear | Yes | Yes | Unclear | Yes | Unclear | Yes | Yes | 6 | Mod |
| 26 | Opoku Agyemang *et al.* 2022a | Yes | Yes | Yes | Yes | Yes | Yes | Yes | Yes | Yes | 9 | High |
| 27 | Opoku Agyemang *et al*. 2022b | Yes | Yes | Yes | Yes | Yes | Yes | Yes | Yes | Yes | 9 | High |
| 28 | Ae-Ngibise *et al.* 2023 | Yes | Yes | Yes | Yes | Yes | Yes | Yes | Yes | Unclear | 8 | High |
| 29 | Agyekum *et al.* 2023 | Unclear | Unclear | Yes | Yes | Yes | Yes | Unclear | Yes | Unclear | 5 | Mod |
| 30 | Anum *et al.* 2023 | Unclear | No | No | Unclear | Unclear | Yes | Unclear | Unclear | Unclear | 1 | Low |
| 31 | Assefa *et al.* 2023 | Yes | Unclear | No | Yes | Unclear | Yes | Yes | Yes | Unclear | 5 | Mod |
| 32 | Dordoye *et al.* 2023 | Yes | Unclear | No | Yes | Yes | Yes | Unclear | Yes | Unclear | 5 | Mod |
| 33 | Nakua *et al.* 2023 | Yes | Yes | Yes | Yes | Yes | Yes | Yes | Yes | Yes | 9 | High |
| 34 | Siakwa *et al.* 2015 | Yes | Unclear | No | Yes | Unclear | Yes | Unclear | Yes | Unclear | 4 | Mod |
| 35 | Kugbey 2022 | Yes | Unclear | No | Yes | Yes | Yes | Unclear | Unclear | Unclear | 4 | Mod |
| 36 | Calys-Tagoe *et al.* 2017 | Yes | Unclear | No | Yes | Yes | Yes | Unclear | No | Yes | 5 | Mod |
| 37 | Fofie *et al.* 2023 | Yes | No | Yes | Yes | Yes | Yes | Unclear | No | Unclear | 5 | Mod |
| 38 | Nutor *et al.* 2024 | Yes | No | Yes | Yes | Unclear | Yes | Yes | Yes | Unclear | 6 | Mod |

Overall quality rating: Low quality; Mod. = Moderate quality; and High quality

# **Appendix F. References of included studies**

1. **Ademola A, Boima V, Odusola A, Agyekum F, Nwafor C and Salako B** (2019) Prevalence and determinants of depression among patients with hypertension: A cross-sectional comparison study in Ghana and Nigeria. *Nigerian Journal of Clinical Practice* **22**(4), 558–565. https://doi.org/10.4103/njcp.njcp_351_18.
2. **Adjepong M, Amoah-Agyei F, Du C, Wang W, Fenton JI and Tucker RM** (2022) Limited negative effects of the COVID-19 pandemic on mental health measures of Ghanaian university students. *Journal of Affective Disorders Reports* **7**, 100306. https://doi.org/10.1016/j.jadr.2021.100306.
3. **Adu MK, Wallace LJ, Lartey KF, Arthur J, Oteng KF, Dwomoh S, Owusu-Antwi R, Larsen-Reindorf R and Agyapong VIO** (2021) Prevalence and correlates of likely major depressive disorder among the adult population in Ghana during the COVID-19 pandemic. *International Journal of Environmental Research and Public Health* **18**(13), 7106. https://doi.org/10.3390/ijerph18137106.
4. **Ae-Ngibise KA, Sakyi L, Adwan-Kamara L, Lund C and Weobong B** (2023) Prevalence of probable mental, neurological and substance use conditions and case detection at primary healthcare facilities across three districts in Ghana: Findings from a cross-sectional health facility survey. *BMC Psychiatry* **23**(1). https://doi.org/10.1186/s12888-023-04775-z.
5. **Agyekum JA, Gyamfi T and Yeboah K** (2023) Depression, poor sleep quality, and diabetic control in type 2 diabetes patients at Sunyani Regional Hospital, Ghana: A case–control study. *Middle East Current Psychiatry* **30**(1), 45. https://doi.org/10.1186/s43045-023-00317-1.
6. **Akpalu J, Yorke E, Ainuson-Quampah J, Balogun W and Yeboah K** (2018) Depression and glycaemic control among type 2 diabetes patients: A cross-sectional study in a tertiary healthcare facility in Ghana. *BMC Psychiatry* **18**(1). https://doi.org/10.1186/s12888-018-1933-2.
7. **Amoako Y, Ackam N, Omuojine J-P, Oppong M, Owusu-Ansah A, Boateng H, Abass K, Amofa G, Ofori E, Boakye Okyere P, Frimpong M, Bailey F, Molyneux D and Phillips R** (2021) Mental health and quality of life burden in Buruli ulcer disease patients in Ghana. *Infectious Diseases of Poverty* **10**. https://doi.org/10.1186/s40249-021-00891-8.
8. **Amu H, Osei E, Kofie P, Owusu R, Bosoka SA, Konlan KD, Kim E, Orish VN, Maalman RS-E, Manu E, Parbey PA, Saah FI, Mumuni H, Appiah PK, Komesuor J, Ayanore MA, Amenuvegbe GK, Kim S, Jung H, Adjuik M, Tarkang EE, Alhassan RK, Donkor ES, Zottor FB, Kweku M, Amuna P, Kim SY and Gyapong JO** (2021) Prevalence and predictors of depression, anxiety, and stress among adults in Ghana: A community-based cross-sectional study. *PLoS ONE* **16**(10), e0258105. https://doi.org/10.1371/journal.pone.0258105.
9. **Anum A, Acquah B and Osei-Tutu A** (2023) Police mental health in ghana: Examining the effects of personality and police rank. *Trends in Psychology*. https://doi.org/10.1007/s43076-023-00302-x.
10. **Arthur-Mensah R, Paintsil GP, Agudu Delali A and Kyei AA** (2022) Mental health outcomes and mental hygiene in the COVID-19 era: A cross-sectional study among healthcare workers from a regional hospital in Ghana. *Psychology Research and Behavior Management* **Volume 15**, 21–30. https://doi.org/10.2147/PRBM.S337740.
11. **Asare-Doku W, Rich JL, Kelly B, Amponsah-Tawiah K and James C** (2021) Mental health and mining: the Ghanaian gold mining story. *International Archives of Occupational and Environmental Health* **94**(6), 1353–1362. https://doi.org/10.1007/s00420-021-01726-7.
12. **Assefa N, Abdullahi YY, Hemler EC, Lankoande B, Madzorera I, Wang D, Ismail A, Chukwu A, Workneh F, Mapendo F, Millogo O, Abubakari SW, Febir LG, Lyatuu I, Dianou K, Baernighausen T, Soura A, Asante KP, Smith E, Vuai S, Worku A, Killewo J, Mwanyika-Sando M, Berhane Y, Sie A, Tajudeen R, Oduola A and Fawzi WW** (2023) COVID-19 preventive practices, psychological distress, and reported barriers to healthcare access during the pandemic among adult community members in Sub-Saharan Africa: A phone survey. *American Journal of Tropical Medicine and Hygiene* **108**(1), 124–136. https://doi.org/10.4269/ajtmh.22-0349.
13. **Awuah RB, de-Graft Aikins A, Dodoo FN-A, Meeks KA, Beune EJ, Klipstein-Grobusch K, Addo J, Smeeth L, Bahendeka SK and Agyemang C** (2019) Psychosocial factors and hypertension prevalence among Ghanaians in Ghana and Ghanaian migrants in Europe: The RODAM study. *Health Psychology Open* **6**(2), 2055102919885752. https://doi.org/10.1177/2055102919885752.
14. **Boateng GO, Doku DT, Enyan NIE, Owusu SA, Aboh IK, Kodom RV, Ekumah B, Quansah R, Boamah SA, Obiri-Yeboah D, Nsabimana E, Jansen S and Armah FA** (2021) Prevalence and changes in boredom, anxiety and well-being among Ghanaians during the COVID-19 pandemic: A population-based study. *BMC Public Health* **21**(1), 985. https://doi.org/10.1186/s12889-021-10998-0.
15. **Boima V, Ademola AD, Odusola AO, Agyekum F, Nwafor CE, Cole H, Salako BL, Ogedegbe G and Tayo BO** (2015) Factors associated with medication nonadherence among hypertensives in Ghana and Nigeria. *International Journal of Hypertension* **2015**. https://doi.org/10.1155/2015/205716.
16. **Calys-Tagoe B, NAH S, CA A and Clegg-Lamptey JN** (2017) Anxiety and depression among breast cancer patients in a tertiary hospital in Ghana. *Postgraduate Medical Journal of Ghana* **6**, 54–8. https://doi.org/10.60014/pmjg.v6i1.112.
17. **Canavan ME, Sipsma HL, Adhvaryu A, Ofori-Atta A, Jack H, Udry C, Osei-Akoto I and Bradley EH** (2013) Psychological distress in Ghana: Associations with employment and lost productivity. *International Journal of Mental Health Systems* **7**(1). https://doi.org/10.1186/1752-4458-7-9.
18. **Danquah J and Mante PK** (2022) Post-illness anxiety, depression and PTSD symptoms in COVID-19 survivors. *International Journal of Mental Health* **51**(2), 131–141. https://doi.org/10.1080/00207411.2022.2028057.
19. **Dordoye E, Dziwornu E, Atisu E and Sarfo JO** (2023) Depressive symptoms among medical students: A cross-sectional study of prevalence and sociodemographic determinants in Ghana. *European Journal of Contemporary Education* **12**, 71–78. https://doi.org/10.13187/ejced.2023.1.71.
20. **Fofie EA, Ekpor E and Akyirem S** (2023) Postpandemic fear of COVID-19, psychological distress, and resilient coping among frontline health workers in Ghana: An analytical cross-sectional study. *Health Science Reports* **6**(10), e1608. https://doi.org/10.1002/hsr2.1608.
21. **Ganu VJ, Boima V, Adjei DN, Yendork JS, Dey ID, Yorke E, Mate-Kole CC and Mate-Kole MO** (2018) Depression and quality of life in patients on long term hemodialysis at a nationalhospital in Ghana: A cross-sectional study. *Ghana Medical Journal* **52**(1), 22–28. https://doi.org/10.4314/gmj.v52i1.5.
22. **Gyasi R, Phillips D and Abass K** (2019) Social support networks and psychological wellbeing in community-dwelling older Ghanaian cohorts. *International Psychogeriatrics* **31**, 1047–1057. https://doi.org/10.1017/S1041610218001539.
23. **Kretchy IA, Owusu-Daaku FT and Danquah SA** (2014) Mental health in hypertension: Assessing symptoms of anxiety, depression and stress on anti-hypertensive medication adherence. *International Journal of Mental Health Systems* **8**(1). https://doi.org/10.1186/1752-4458-8-25.
24. **Kugbey N** (2022) Comorbid anxiety and depression among women receiving care for breast cancer: analysis of prevalence and associated factors. *African Health Sciences* **22**(3), 166–172. https://doi.org/10.4314/ahs.v22i3.19.
25. **Kugbey N, Nortu TA, Akpalu B, Ayanore MA and Zotor FB** (2018) Prevalence of geriatric depression in a community sample in Ghana: Analysis of associated risk and protective factors. *Archives of Gerontology and Geriatrics* **78**, 171–176. https://doi.org/10.1016/j.archger.2018.06.014.
26. **Nakua EK, Amissah J, Tawiah P, Barnie B, Donkor P and Mock C** (2023) The prevalence and correlates of depression among older adults in greater Kumasi of the Ashanti region. *BMC Public Health* **23**(1), 763. https://doi.org/10.1186/s12889-023-15361-z.
27. **Nutor JJ, Alhassan RK, Thompson RGA, Asakitogum DA, Duah HO, Ritchwood TD, Nkosi N, Klaas N, Agyemang SO and Gyamerah AO** (2024) Depression and its associated factors among people living with HIV in the Volta region of Ghana. *PLOS Mental Health* **1**(1), e0000035. https://doi.org/10.1371/journal.pmen.0000035.
28. **Nuvey FS, Kreppel K, Nortey PA, Addo-Lartey A, Sarfo B, Fokou G, Ameme DK, Kenu E, Sackey S, Addo KK, Afari E, Chibanda D and Bonfoh B** (2020) Poor mental health of livestock farmers in Africa: A mixed methods case study from Ghana. *BMC Public Health* **20**(1). https://doi.org/10.1186/s12889-020-08949-2.
29. **Ofori AA, Osarfo J, Agbeno EK, Manu DO and Amoah E** (2021) Psychological impact of COVID-19 on health workers in Ghana: A multicentre, cross-sectional study. *SAGE Open Medicine* **9**, 20503121211000919. https://doi.org/10.1177/20503121211000919.
30. **Ofori-Atta A, Reynolds NR, Antwi S, Renner L, Nichols JS, Lartey M, Amissah K, Tettey JK, Alhassan A, Ofori IP, Catlin AC, Gan G, Kyriakides TC and Paintsil E** (2019) Prevalence and correlates of depression among caregivers of children living with HIV in Ghana: Findings from the Sankofa pediatric disclosure study. *AIDS Care - Psychological and Socio-Medical Aspects of AIDS/HIV* **31**(3), 283–292. https://doi.org/10.1080/09540121.2018.1537463.
31. **Omuojine J-P, Nguah SB, Ayisi-Boateng NK, Sarfo FS and Ovbiagele B** (2022) Contemporary prevalence and predictors of anxiety among patients living with HIVAIDS in Ghana. *Ghana Medical Journal* **56**(3), 169–175.
32. **Opoku Agyemang S, Ninnoni JP and Enyan NIE** (2022a) Prevalence and determinants of depression, anxiety and stress among psychiatric nurses in Ghana: A cross-sectional study. *BMC Nursing* **21**(1), 1–11.
33. **Opoku Agyemang S, Ninonni J, Bennin L, Agyare E, Gyimah L, Senya K, Birikorang E, Quarshie EN-B, Baddoo NA, Addo SA and Obiri-Yeboah D** (2022b) Prevalence and associations of depression, anxiety, and stress among people living with HIV: A hospital-based analytical cross-sectional study. *Health Science Reports* **5**(5). https://doi.org/10.1002/hsr2.754.
34. **Oppong Asante K and Andoh-Arthur J** (2015) Prevalence and determinants of depressive symptoms among university students in Ghana. *Journal of Affective Disorders* **171**, 161–166. https://doi.org/10.1016/j.jad.2014.09.025.
35. **Saah FI, Amu H and Kissah-Korsah K** (2021) Prevalence and predictors of work-related depression, anxiety, and stress among waiters: A cross-sectional study in upscale restaurants. *PLoS ONE* **16**(4 April). https://doi.org/10.1371/journal.pone.0249597.
36. **Shaikh A, Peprah E, Mohamed RH, Asghar A, Andharia NV, Lajot NA and Qureshi MFH** (2021) COVID-19 and mental health: A multi-country study—the effects of lockdown on the mental health of young adults. *Middle East Current Psychiatry* **28**(1), 51. https://doi.org/10.1186/s43045-021-00116-6.
37. **Siakwa M, Okanlawon FA, Druye AA, Ankobil A, Aniweh Y and Dzah S** (2015) Prevalence of psychiatric disorders in HIV patients in the Central Region of Ghana. *Journal of Community Medicine and Primary Health Care* **27**(1), 79–85.
38. **Swaray SM, Tetteh J, Ekem-Ferguson G, Awinibuno IAN, Adu-Gyasi D, Acheampong F and Yawson AE** (2021) Psychological distress amongst medical laboratory professionals involved in covid-19-related duties: A nationally stratified cross-sectional survey, Ghana. *Inquiry: A Journal of Medical Care Organization, Provision and Financing* **58**, 00469580211067479. https://doi.org/10.1177/00469580211067479.

# **Appendix G. All reasons for exclusion (N= 234)**

| **Study** | **Overall category (s)** | **Reason(s) for exclusion** |
| --- | --- | --- |
| Abass *et al.* 2022 [1] | Ineligible outcome | Does not report the prevalence of anxiety and depressive disorders and symptoms, and/or symptoms of psychological distress |
| Abass *et al.* 2023 [2] | Ineligible outcome | Does not report the prevalence of anxiety and depressive disorders and symptoms, and/or symptoms of psychological distress |
| Abbey *et al.* 2021 [3] | Ineligible outcome | Does not report the prevalence of anxiety and depressive disorders and symptoms, and/or symptoms of psychological distress |
| Abbey and Nasidi 2023 [4] | Ineligible study type | Qualitative study |
| Abeasi 2020 [5] | Ineligible outcome | Does not report the prevalence of anxiety and depressive disorders and symptoms, and/or symptoms of psychological distress |
| Aberese-Ako *et al.* 2022 [6] | Ineligible assessment method | Does not provide information on validity and/or reliability |
| Abunyewah *et al.* 2024 [7] | Ineligible outcome | Does not report the prevalence of anxiety and depressive disorders and symptoms, and/or symptoms of psychological distress |
| Achore and Bisung 2022 [8] | Ineligible assessment method | Measured psycho-emotional distress using one question |
| Acolatse 2020 [9] | Ineligible outcome | Does not report the prevalence of anxiety and depressive disorders and symptoms, and symptoms of psychological distress |
| Adam *et al.* 2023 [10] | Ineligible population | Focused on adults accused of witchcraft and living in alleged witches’ camps |
| Addai and Andrees 2014 [11] | Ineligible study type | Book chapter |
| Adejumo *et al.* 2019 [12] | Ineligible context | Targeted adults living in Nigeria |
| Adhvaryu *et al.* 2019 [13] | Repeated dataset | Used the same dataset that was used in one of the included studies |
| Adjaye-Gbewonyo *et al.* 2019a [14] | Ineligible outcome | Does not report the prevalence of anxiety and depressive disorders and symptoms, and/or symptoms of psychological distress  Results based on secondary analysis (SAGE dataset) |
| Adjaye-Gbewonyo *et al.* 2020 [15] | Repeated dataset ^a^  Ineligible outcome ^b^ | Results based on secondary analysis (repeated SAGE dataset)  Does not report the prevalence of anxiety and depressive disorders and symptoms, and/or symptoms of psychological distress |
| Adjaye-Gbewonyo *et al.* 2019b [16] | Repeated dataset ^a^  Ineligible assessment method ^b^ | Results based on secondary analysis (repeated SAGE dataset)  Problems with measurement of depression: e.g., participants were classified as having depression if they self-reported treatment or if in the past 12 months they met the ICD-10 criteria for mild depressive episode based on an algorithm developed from reported symptoms. |
| **Adjei *et al.* 2015** [17] | Ineligible study type | Scientific and/or conference abstract |
| Adjei *et al.* 2019 [18] | Repeated dataset | Analysed same dataset that was used in an included study |
| Adjorlolo *et al.* 2017 [19] | Ineligible outcome | Does not report the prevalence of anxiety and depressive disorders and symptoms, and/or symptoms of psychological distress |
| Adzika *et al.* 2016 [20] | Ineligible study type | Conference abstract |
| Adzika *et al.* 2017 [21] | Ineligible study type ^a^  Ineligible outcome ^b^ | Conference abstract  Reported prevalence of sickle cell disease rather than prevalence of anxiety and depression disorders and symptoms, and/or symptoms of psychological distress |
| Ae-Ngibise *et al.* 2017 [22] | Ineligible assessment method ^a^  Ineligible outcome ^b^ | Focused on adult with mental and neurological disorders assessed via psychiatric case register  Does not report the prevalence of anxiety and depressive disorders and symptoms, and/or symptoms of psychological distress |
| Agyei *et al.* 2022 [23] | Ineligible assessment method | Does not provide information on validity and/or reliability |
| Ahorsu *et al.* 2021 [24] | Ineligible outcome | Does not report the prevalence of anxiety and depressive disorders and symptoms, and/or symptoms of psychological distress |
| Ahulu *et al.* 2020 [25] | Ineligible population ^a^  Ineligible outcome ^b^ | Focused on adolescents aged 13-19 years  Does not provide separate prevalence data for adult participants aged ≥ 18 years |
| Akanko *et al.* 2023 [26] | Ineligible assessment method | Does not provide information on validity and/or reliability |
| Alhassan *et al.* 2014 [27] | Ineligible population | Focused on adults with other cormobid chronic physical condition (i.e., Ghanaian infertile women) |
| Al-Riyami *et al.* 2022 [28] | Ineligible assessment method | Measured anxiety and depressive symptoms, and/or symptoms of psychological distress using one question |
| Amankwah-Poku *et al.* 2020 [29] | Ineligible outcome | Does not report the prevalence of anxiety and depressive disorders and symptoms, and/or symptoms of psychological distress |
| Ambugo 2014 [30] | Ineligible outcome | Does not report the prevalence of anxiety and depressive disorders and symptoms, and/or symptoms of psychological distress |
| Amegbor *et al.* 2020 [31] | Repeated dataset ^a^  Ineligible outcome ^b^ | Results based on secondary analysis (repeated SAGE dataset)  Does not report provide separate depression prevalence data for Ghanaian participants |
| Amegbor *et al.* 2021 [32] | Repeated dataset ^a^  Ineligible outcome ^b^ | Prevalence data based on secondary analysis (repeated SAGE wave 1 and 2 datasets) |
| Amissah and Nyarko 2020 [33] | Ineligible condition | Measured symptoms of hopelessness depression |
| Amissah *et al.* 2023 [34] | Ineligible outcome | Does not report the prevalence of anxiety and depressive disorders and symptoms, and/or symptoms of psychological distress |
| Amissah *et al.* 2022 [35] | Ineligible study type | Conference abstract |
| Amoah *et al.* 2022 [36] | Ineligible outcome | Does not report the prevalence of anxiety and depression disorders and symptoms, and/or symptoms of psychological distress |
| Amoah *et al.* 2023 [37] | Ineligible outcome | Does not report the prevalence of anxiety and depressive disorders and symptoms, and/or symptoms of psychological distress |
| Anim *et al.* 2016 [38] | Ineligible outcome | Focused on adults with sickle cell disease  Prevalence of psychological distress reported as mean and standard deviation |
| Anim-Sampong *et al.* 2021 [39] | Ineligible assessment method | Does not provide information on validity and/or reliability |
| Ansah *et al.* 2023 [40] | Ineligible outcome | Does not report the prevalence of anxiety and depressive disorders and symptoms, and/or symptoms of psychological distress |
| Appiah-Poku *et al.* 2004 [41] | Ineligible outcome | Does not report the prevalence of anxiety and depressive disorders and symptoms, and/or symptoms of psychological distress |
| Arku *et al.* 2011 [42] | Ineligible assessment method | Measured anxiety and depressive symptoms, and/or symptoms of psychological distress using one question |
| Arokiasamy *et al.* 2015 [43] | Repeated dataset ^a^  Ineligible outcome ^b^ | Results based on secondary analysis (repeated dataset)  Does not report the prevalence of anxiety and depressive disorders and symptoms, and/or symptoms of psychological distress |
| Asante 2012 [44] | Ineligible outcome | Problems with prevalence estimates (prevalence data could not be obtained via study authors) |
| Asante *et al.* 2023 [45] | Ineligible assessment method | Does not provide information on validity and/or reliability |
| Ashdown-Franks *et al.* 2019 [46] | Repeated dataset ^a^  Ineligible outcome ^b^ | Results based on secondary analysis (repeated dataset)  Does not report the prevalence of anxiety and depressive disorders and symptoms, and/or symptoms of psychological distress |
| Asiedu *et al.* 2018 [47] | Ineligible outcome | Does not report the prevalence of anxiety and depressive disorders and symptoms, and/or symptoms of psychological distress |
| Asiedu *et al.* 2020 [48] | Ineligible outcome | Does not report the prevalence of anxiety and depressive disorders and symptoms, and/or symptoms of psychological distress |
| Asiwome 2020 [49] | Ineligible study type | Thesis and dissertation |
| Asoogo *et al.* 2018 [50] | Ineligible study type | Conference abstract |
| Atefoe and Kugbey 2018 [51] | Ineligible outcome | Does not report the prevalence of anxiety and depressive disorders and symptoms, and/or symptoms of psychological distress |
| Atefoe *et al.* 2017 [52] | Ineligible outcome | Does not report the prevalence of anxiety and depressive disorders and symptoms, and/or symptoms of psychological distress |
| Atuoye and Luginaah 2017 [53] | Ineligible outcome | Does not report the prevalence of anxiety and depressive disorders and symptoms, and/or symptoms of psychological distress |
| Awanyo *et al.* 2017 [54] | Ineligible outcome | Does not report the prevalence of anxiety and depressive disorders and symptoms, and/or symptoms of psychological distress |
| Ayisi-Boateng *et al.* 2022 [55] | Repeated dataset | Used the same dataset that was used in one of the included studies |
| Azure 2011 [56] | Ineligible condition | Measured course anxiety |
| Babagoli *et al.* 2024 [57] | Ineligible assessment method | Multiple measurement, potentially skewed prevalence outcomes |
| Benneh *et al.* 2014 [58] | Ineligible study type | Qualitative study design |
| Bettmann *et al.* 2019 [59] | Ineligible outcome | Does not report the prevalence of anxiety and depressive disorders and symptoms, and/or symptoms of psychological distress |
| Bhan *et al.* 2020 [60] | Ineligible assessment method ^a^  Repeated dataset ^b^ | Feeling depressed and anxiety were assessed by one question  Results based on secondary analysis (repeated SAGE dataset) |
| Bisung *et al.* 2018 [61] | Ineligible assessment method | Measured depressive symptoms using a single question |
| Błachnio *et al.* 2022 [62] | Ineligible outcome | Does not report the prevalence of anxiety and depressive disorders and symptoms, and/or symptoms of psychological distress |
| Blankson *et al.* 2021 [63] | No access to eligibility data | No access to eligibility data |
| Boadi-Kusi *et al.* 2023 [64] | Ineligible outcome | Does not report the prevalence of anxiety and depressive disorders and symptoms, and/or symptoms of psychological distress |
| Boima *et al.* 2020 [65] | Ineligible assessment method ^a^  Repeated dataset ^b^ | Problems with depressive symptoms measurement: e.g., first participant had to yes to either one of 3 questions on depressive episode, before proceeding to answering 15 standard questions  Results based on secondary analysis (repeated SAGE dataset) |
| Bonful and Anum 2019 [66] | Ineligible outcome | Results based on secondary analysis  Does not report the prevalence of anxiety and depressive disorders and symptoms, and/or symptoms of psychological distress |
| Bonsu *et al.* 2019 [67] | Ineligible outcome | Does not report the prevalence of anxiety and depressive disorders and symptoms, and/or symptoms of psychological distress |
| Boyce *et al.* 2009 [68] | Ineligible outcome | Targeted outcomes related to mental health progress  Does not report the prevalence of anxiety and depressive disorders and symptoms, and/or symptoms of psychological distress |
| Brinda *et al.* 2016 [69] | Repeated dataset ^a^  Ineligible assessment method ^b^ | Results based on secondary analysis (repeated SAGE data)  Problems with measurement (e.g., an algorithm was developed to derive ICD-10 diagnoses of depression from SAGE data) |
| Cabello *et al.* 2017 [70] | Ineligible assessment method ^a^  Repeated dataset ^b^ | Problems with measurement., SAGE wave 0 and 1 datasets were used to categorised incident depression group and persistent depression group  Results based on secondary analysis (repeated SAGE dataset) |
| Chan *et al.* 2017a [71] | Ineligible assessment method ^a^  Repeated dataset ^b^ | Problems with measurement (e.g., participants reported whether they had been clinically diagnosed with depression, and further asked if they experienced symptoms of depression based on DSM-IV. There no information about the use of structured clinical interview for DSM-4)  Results based on secondary analysis (repeated SAGE dataset) |
| Chan *et al.* 2017b [72] | Ineligible study type | Poster abstract |
| Chan *et al.* 2015 [73] | Ineligible outcome ^a^  Repeated dataset ^b^ | Does not report the prevalence of anxiety and depressive disorders and symptoms, and/or symptoms of psychological distress  Results based on secondary analysis (repeated SAGE data set) |
| Chirwa *et al.* 2020 [74] | Repeated dataset ^a^  Ineligible outcome ^b^ | Repeated dataset  Does not report the prevalence of anxiety and depressive disorders and symptoms, and/or symptoms of psychological distress |
| Clegg-Lamptey *et al.* 2009 [75] | Ineligible assessment method | Measured anxiety symptom as a reaction to breast cancer diagnosis |
| Cole *et al.* 2015 [76] | Ineligible outcome | Does not report the prevalence of anxiety and depressive disorders and symptoms, and/or symptoms of psychological distress |
| Dadzie *et al.* 2023 [77] | Ineligible outcome | Does not report the prevalence of anxiety and depressive disorders and symptoms, and/or symptoms of psychological distress |
| Darko *et al.* 2024 [78] | Ineligible outcome | Does not report the prevalence of anxiety and depressive disorders and symptoms, and/or symptoms of psychological distress |
| de Menil *et al.* 2012 [79] | Ineligible outcome | Does not report the prevalence of anxiety and depressive disorders and symptoms, and/or symptoms of psychological distress |
| Dey *et al.* 2022 [80] | Ineligible outcome | Does not report the prevalence of anxiety and depressive disorders and symptoms, and/or symptoms of psychological distress |
| Doglikuu *et al.* 2021a [81] | Ineligible outcome | Does not report the prevalence of anxiety and depressive disorders and symptoms, and/or symptoms of psychological distress |
| Doglikuu *et al.* 2021b [82] | Ineligible outcome | Does not report the prevalence of anxiety and depressive disorders and symptoms, and/or symptoms of psychological distress |
| Donnir and Asare-Doku 2021 [83] | Ineligible study type | Conference abstract |
| Donnir and Asare-Doku 2023 [84] | Ineligible population | Focused on prisoners |
| Duda *et al.* 2011 [85] | Ineligible condition | Measured symptoms of menopause |
| Durizzo *et al.* 2022 [86] | Ineligible assessment method | Measured feeling depressed on a likert scale, and indicated that this was a rudimentary indicator of depression and might be less accurate |
| Dzator 2013 [87] | No access to eligibility criteria | No access to eligibility data |
| Ed-Bansah *et al.* 2023 [88] | Ineligible outcome | Does not report the prevalence of anxiety and depressive disorders and symptoms, and/or symptoms of psychological distress |
| Ekem-Ferguson *et al.* 2017 [89] | Ineligible condition | Targeted diabetes-related depression |
| Ekem-Ferguson *et al.* 2022 [90] | Ineligible outcome | Does not report the prevalence of anxiety and depressive disorders and symptoms, and/or symptoms of psychological distress |
| Ephraim *et al.* 2021 [91] | Ineligible condition | Targeted diabetes-related distress |
| Fernandez-Nino *et al.* 2019 [92] | Ineligible outcome ^a^  Repeated dataset ^b^ | Does not report the prevalence of anxiety and depressive disorders and symptoms, and/or symptoms of psychological distress  Results based on secondary analysis (repeated SAGE dataset) |
| Fernandez-Nino *et al.* 2018 [93] | Repeated dataset | Repeated dataset |
| Folayan *et al.* 2022a [94] | Ineligible context ^a^  Ineligible outcome ^b^ | Targeted West Africa  Does not report provide separate depression prevalence data for Ghanaian participants |
| Folayan *et al.* 2022b [95] | Ineligible context ^a^  Ineligible outcome ^b^ | Targeted sub region in sub-Saharan Africa  Does not report provide separate depression prevalence data for Ghanaian participants |
| Frimpong *et al.* 2022 [96] | Ineligible outcome | Reported prevalence of work-related anxiety and depressive symptoms  Prevalence estimates also included pre-existing anxiety and depression diagnosis given by a friend, family member, medical staff, workmate and self |
| Garin *et al.* 2016 [97] | Repeated dataset  Ineligible assessment method | Results based on secondary analysis (repeated dataset)  Measured depressive symptoms based on combined method consisting of self-reported physician’s diagnosis and symptom-based algorithms |
| Gehlich *et al.* 2019 [98] | Ineligible assessment method ^a^  Ineligible outcome ^b^ | Does not use a psychometrically tested depression assessment procedure  Does not report provide separate depression prevalence data for Ghanaian participants  Results based on secondary analysis (repeated dataset) |
| Glozah *et al.* 2018 [99] | Ineligible assessment method ^a^  Ineligible outcome ^b^ | Mental distress measured using three indicators  No information on tool validity or reliability  Does not report provide separate depression prevalence data for Ghanaian participants |
| Greif and Nii-Amoo Dodoo 2015 [100] | Ineligible assessment method | Measured depressive symptoms using only one question “how often the respondent felt depressed in the last month” |
| Gyasi 2019 [101] | Ineligible outcome | Does not report the prevalence of anxiety and depressive disorders and symptoms, and/or symptoms of psychological distress |
| Gyasi *et al.* 2020a [102] | Repeated dataset | Repeated dataset |
| Gyasi *et al.* 2022 [103] | Repeated dataset | Repeated dataset |
| Gyasi *et al.* 2019a [104] | Repeated dataset | Repeated dataset |
| Gyasi *et al.* 2023a [105] | Repeated dataset | Repeated dataset |
| Gyasi *et al.* 2023b [106] | Repeated dataset | Repeated dataset |
| Gyasi *et al.* 2020b [107] | Repeated dataset | Repeated dataset |
| Gyasi *et al.* 2020c [108] | Repeated dataset | Repeated dataset |
| Gyasi and Phillips 2020 [109] | Repeated dataset | Repeated dataset |
| Gyasi *et al.* 2019b [110] | Repeated dataset | Repeated dataset |
| Hommey *et al.* 2020 [111] | Ineligible outcome | Does not report the prevalence of anxiety and depressive disorders and symptoms, and/or symptoms of psychological distress |
| Huang *et al.* 2018 [112] | Ineligible outcome | Does not report the prevalence of anxiety and depressive disorders and symptoms, and/or symptoms of psychological distress |
| Huang *et al.* 2020 [113] | Repeated dataset ^a^  Ineligible outcome ^b^ | Result based on secondary analysis (repeated SAGE dataset)  Does not report the prevalence of anxiety and depressive disorders and symptoms, and/or symptoms of psychological distress |
| Ibrahim *et al.* 2016 [114] | Ineligible condition | Targeted anxiety symptoms influenced by erratic electricity supply |
| Ibrahim *et al.* 2015 [115] | Ineligible population | Focused on prisoners |
| Jacob *et al.* 2019 [116] | Repeated dataset ^a^  Ineligible outcome ^b^ | Result based on secondary analysis (repeated SAGE dataset)  Measured depressive symptoms based on combined method consisting of self-reported physician’s diagnosis and symptom-based algorithms |
| Jacob *et al.* 2023[117] | Repeated dataset ^a^  Ineligible outcome ^b^ | Result based on secondary analysis (repeated SAGE dataset)  Does not report provide separate depression prevalence data for Ghanaian participants |
| Judge *et al.* 2021 [118] | Ineligible study type | Conference abstract |
| Kaburi *et al.* 2019 [119] | Ineligible condition | Measured occupational stress |
| Kawada 2018 [120] | Ineligible study type | Letter to the Editor |
| Kennedy *et al.* 2023 [121] | Ineligible outcome | Does not report the prevalence of anxiety and depressive disorders and symptoms, and/or symptoms of psychological distress |
| Khumalo *et al.* 2022 [122] | Ineligible outcome | Does not report the prevalence of anxiety and depressive disorders and symptoms, and/or symptoms of psychological distress |
| komesuor and Meyer-Weitz 2023 [123] | Ineligible outcome | Problems with prevalence estimates |
| Kploanyi *et al.* 2020 [124] | Ineligible assessment method ^a^  Ineligible outcome ^b^ | Depressive symptom was measured using self-administered questionnaire adapted from the National Institute for Occupational safety and Health; and CES-D scale employed in Generic Job Stress Questionnaire  Does not report the prevalence of anxiety and depressive disorders and symptoms, and/or symptoms of psychological distress |
| Kpotosu and Pinkrah 2023 [125] | Ineligible outcome | Does not report the prevalence of anxiety and depressive disorders and symptoms, and/or symptoms of psychological distress |
| Krass *et al.* 2014 [126] | Ineligible study type | Conference abstract |
| Kretchy 2022 [127] | Ineligible study type | Commentary |
| Kretchy *et al.* 2020 [128] | Repeated dataset | Repeated dataset |
| Kretchy *et al.* 2018 [129] | Ineligible outcome | Does not report the prevalence of anxiety and depressive disorders and symptoms, and/or symptoms of psychological distress |
| Kretchy *et al.* 2015 [130] | Repeated dataset | Repeated dataset |
| Kugbey *et al.* 2019 [131] | Ineligible outcome | Does not report the prevalence of anxiety and depressive disorders and symptoms, and/or symptoms of psychological distress |
| Kugbey *et al.* 2020 [132] | Ineligible outcome | Does not report the prevalence of anxiety and depressive disorders and symptoms, and/or symptoms of psychological distress |
| Kunna *et al.* 2017 [133] | Repeated dataset | Results based on secondary analysis (repeated dataset) |
| Kushitor *et al.* 2018 [134] | Repeated dataset | Results based on secondary analysis (repeated dataset) |
| Kwawukume *et al.* 1993 [135] | Ineligible outcome | Does not report the prevalence of anxiety and depressive disorders and symptoms, and/or symptoms of psychological distress |
| Kyei 2017 [136] | Ineligible study type | Dissertation |
| Kyei *et al.* 2020 [137] | Ineligible assessment method ^a^  Ineligible outcome ^b^ | No information on validity and reliability of modified tool  Does not report the prevalence of anxiety and depressive disorders and symptoms, and/or symptoms of psychological distress |
| Lambert *et al.* 2017 [138] | Repeated dataset ^a^  Ineligible outcome ^b^ | Results based on secondary analysis (repeated SAGE dataset)  Does not report the prevalence of anxiety and depressive disorders and symptoms, and/or symptoms of psychological distress |
| Langsi *et al.* 2021 [139] | Ineligible context ^a^  Ineligible outcome ^b^ | Targeted sub-Saharan Africa  Does not report provide separate depression prevalence data for Ghanaian participants |
| Lawrence *et al.* 2022 [140] | Ineligible population | Focused on families affected by a maternal mortality |
| Lestari 2019 [141] | Repeated dataset ^a^  Ineligible outcome ^b^ | Results based on secondary analysis (repeated SAGE dataset)  Participants who reported taking any medication or treatment for depression were also categorised as having depression  Does not report the prevalence of anxiety and depressive disorders and symptoms, and/or symptoms of psychological distress |
| Li *et al.* 2022 [142] | Repeated dataset | Results based on secondary analysis (repeated SAGE dataset) |
| Lin and Okyere 2020 [143] | Ineligible outcome | Does not report the prevalence of anxiety and depressive disorders and symptoms, and/or symptoms of psychological distress |
| Lin *et al.* 2017 [144] | Repeated dataset ^a^  Ineligible outcome ^b^ | Results based on secondary analysis (repeated dataset)  Does not report the prevalence of anxiety and depressive disorders and symptoms, and/or symptoms of psychological distress |
| Lloyd-Sherlock *et al.* 2019 [145] | Repeated dataset | Results based on secondary analysis (repeated dataset) |
| Lotfaliany *et al.* 2019 [146] | Repeated dataset ^a^  Ineligible outcome ^b^ | Results based on secondary analysis (repeated dataset)  Reported age specific and age standardised prevalence (percentages) of depression |
| Luo *et al.* 2020 [147] | Repeated dataset ^a^  Ineligible outcome | Results based on secondary analysis (repeated dataset)  Does not report provide separate depression prevalence data for Ghanaian participants |
| Majodina and Johnson 1983 [148] | Ineligible outcome | Does not report the prevalence of anxiety and depressive disorders and symptoms, and/or symptoms of psychological distress |
| Marza Florensa *et al.* 2019 [149] | Repeated dataset ^a^  Ineligible outcome ^b^ | Repeated dataset  Does not report the prevalence of anxiety and depressive disorders and symptoms, and/or symptoms of psychological distress |
| Muruthi *et al.* 2022 [150] | Repeated dataset ^a^  Ineligible assessment method ^b^ | Results based on secondary analysis (repeated dataset)  Measured depressed affect using one question |
| **Naab *et al*. 2011 [151]** | Ineligible study type | Dissertation |
| Naab *et al.* 2021 [152] | Ineligible outcome | Measured fertility-related anxiety and depressive symptoms  Does not report the prevalence of anxiety and depressive disorders and symptoms, and/or symptoms of psychological distress |
| Nkyi *et al.* 2021 [153] | Ineligible outcome | Does not report the prevalence of anxiety and depressive disorders and symptoms, and/or symptoms of psychological distress |
| Nkyi and Baaba 2024 [154] | Ineligible condition | Measured COVID-19-related anxiety |
| Nonterah *et al.* 2015 [155] | Ineligible outcome | Does not report the prevalence of anxiety and depressive disorders and symptoms, and/or symptoms of psychological distress |
| Nonterah *et al.* 2023 [156] | Ineligible outcome | Does not report the prevalence of anxiety and depressive disorders and symptoms, and/or symptoms of psychological distress |
| Northuis and Ghazi 2020 [157] | Ineligible study type | Conference abstract |
| Nunfam 2021 [158] | Ineligible condition | Targeted occupational health stress |
| Nutakor *et al.* 2020 [159] | Repeated dataset | Results based on secondary analysis (repeated dataset) |
| Nutor *et al.* 2023 [160] | Repeated dataset | Repeated dataset |
| Nwakasi *et al.* 2021 [161] | Repeated dataset | Results based on secondary analysis (repeated dataset) |
| Nyarko *et al.* 2021 [162] | Repeated dataset | Results based on secondary analysis (repeated dataset) |
| Nyundo *et al.* 2020 [163] | Ineligible population | Targeted adolescents aged 10-19 years and does not provide separate prevalence data for adult participants aged ≥ 18 years |
| Obo *et al.* 2021 [164] | Ineligible outcome | Does not report the prevalence of anxiety and depressive disorders and symptoms, and/or symptoms of psychological distress |
| Ocansey *et al.* 2021 [165] | Ineligible assessment method | Different cutoffs for symptom severity level |
| **Ofori-Atta *et al.* 2014 [166]** | Ineligible study type | Conference abstract |
| Ojagbemi *et al.* 2022 [167] | Ineligible context ^a^  Ineligible outcome ^b^ | Targeted both Ghana and Nigeria but does not provide separate data for Ghanaian participants |
| Ojagbemi *et al.* 2017 [168] | Ineligible context | Targeted sub-Saharan Africa |
| Opoku Mensah *et al.* 2017 [169] | Ineligible population ^a^  Ineligible outcome ^b^ | Ineligible population  Problems with prevalence estimates |
| Oppong Asante *et al.* 2015 [170] | Ineligible population ^a^  Ineligible outcome ^b^ | Targeted homeless children and adolescents  Does not report the prevalence of anxiety and depressive disorders and symptoms, and/or symptoms of psychological distress |
| Oppong *et al.* 2021 [171] | Ineligible population | Focused on adults with other comorbid chronic physical condition (i.e., reproductive-aged women presenting with gynaecological disorders) |
| Opuni *et al.* 2023 [172] | Ineligible condition | Targeted health needs and non-communicable diseases female head porters |
| Osman *et al.* 2023 [173] | Ineligible condition  Ineligible outcome | Measured depression levels per specific damaged household item  Does not report the prevalence of anxiety and depressive disorders and symptoms, and/or symptoms of psychological distress |
| Oti-Boadi *et al.* 2022 [174] | Ineligible outcome | Does not report the prevalence of anxiety and depressive disorders and symptoms, and/or symptoms of psychological distress |
| Owoo and Lambon-Quayefio 2020 [175] | Ineligible study type | Qualitative study design |
| Owusu-Ansah *et al.* 2020 [176] | Ineligible population ^a^  Ineligible outcome ^b^ | Targeted participants aged between 9-32 years  Does not report separate prevalence data for adult participants aged ≥ 18 years |
| Owusu-Ansah *et al.* 2023 [177] | Ineligible outcome | Does not report the prevalence of anxiety and depressive disorders and symptoms, and/or symptoms of psychological distress |
| Owusu-Ansah and Nkrumah 2017 [178] | Ineligible outcome | Does not report the prevalence of anxiety and depressive disorders and symptoms, and/or symptoms of psychological distress |
| Panaite and Cohen 2023 [179] | Repeated dataset ^a^  Ineligible outcome ^b^ | Results based on secondary analysis (repeated SAGE dataset)  Does not report the prevalence of anxiety and depressive disorders and symptoms, and/or symptoms of psychological distress |
| Peele and Wolf 2020 [180] | Ineligible outcome | Does not report the prevalence of anxiety and depressive disorders and symptoms, and/or symptoms of psychological distress |
| Peele and Wolf 2021 [181] | Ineligible outcome | Does not report the prevalence of anxiety and depressive disorders and symptoms, and/or symptoms of psychological distress |
| Peele *et al.* 2023 [182] | Ineligible outcome | Does not report the prevalence of anxiety and depressive disorders and symptoms, and/or symptoms of psychological distress |
| Quansah *et al.* 2022a [183] | Ineligible condition ^a^  Ineligible assessment method ^b^ | Targeted COVID-19-related anxiety symptoms  Adapted the Beck’s anxiety scale, and only items on the non-clinical symptoms of anxiety were selected for use. Further, there was no information about the validity of adapted scale |
| Quansah *et al.* 2022b [184] | Ineligible condition ^a^  Ineligible assessment method ^b^ | Targeted COVID-19-related anxiety symptoms  Adapted the Beck’s anxiety scale, and only items on the non-clinical symptoms of anxiety were selected for use. Further, there was no information about the validity of adapted scale |
| Quansah *et al.* 2022c [185] | Ineligible condition ^a^  Ineligible assessment method ^b^ | Targeted COVID-19-related anxiety symptoms  Adapted the Beck’s anxiety scale, and only items on the non-clinical symptoms of anxiety were selected for use. Further, there was no information about the validity of adapted scale |
| Quashie *et al.* 2019 [186] | Repeated dataset | Results based on secondary analysis (repeated SAGE dataset) |
| Radcliffe *et al.* 2020 [187] | Ineligible outcome | Does not report the prevalence of anxiety and depressive disorders and symptoms, and/or symptoms of psychological distress |
| Ramsoomar *et al.* 2023 [188] | Ineligible outcome | Results based on pooled analysis five dataset |
| Sackey and Sanda 2009 [189] | Ineligible outcome | Does not report the prevalence of anxiety and depressive disorders and symptoms, and/or symptoms of psychological distress |
| Sackey *et al.* 2018 [190] | Ineligible study type | Conference abstract |
| Saeed and Wemakor 2019 [191] | Ineligible condition | Target maternal/postnatal depression |
| Sakyi and Johnson 2022 [192] | Ineligible condition | Target COVID-19 related dysfunctional anxiety |
| Salinas-Rodríguez *et al.* 2020 [193] | Repeated dataset | Results based on secondary analysis (repeated SAGE dataset) |
| Sarfo *et al.* 2019 [194] | Ineligible population ^a^  Ineligible study type ^b^ | Targeted adults >16 years with neurologic disorders  Results based on secondary analysis |
| Sarfo *et al.* 2017a [195] | Ineligible outcome | Does not report the prevalence of anxiety and depressive disorders and symptoms, and/or symptoms of psychological distress |
| Sarfo *et al.* 2017b [196] | Ineligible outcome | Does not report the prevalence of anxiety and depressive disorders and symptoms, and/or symptoms of psychological distress |
| Sarfo *et al.* 2017c [197] | No access to eligibility data | No access to eligibility data |
| Sarfo *et al.* 2017d [198] | Ineligible outcome | Does not report the prevalence of anxiety and depressive disorders and symptoms, and/or symptoms of psychological distress |
| Sarfo and Mate-Kole 2014 [199] | No access to eligibility data | No access to eligibility data |
| Schaefer *et al.* 2007 [200] | Ineligible context | Targeted West Africa but does not provide separate data for Ghanaian participants |
| Selvamani *et al.* 2022 [201] | Repeated dataset | Results based on secondary analysis (repeated SAGE dataset) |
| Shrum *et al.* 2021 [202] | Ineligible outcome | Prevalence of anxiety and depressive symptoms reported as means and standard deviation |
| Shupler *et al.* 2022 [203] | Ineligible condition | Targeted mental health-related quality of life |
| Simiyu *et al.* 2022 [204] | Repeated dataset | Results based on secondary analysis (repeated SAGE dataset) |
| Sipsma *et al.* 2013 [205] | Repeated dataset | Repeated dataset |
| Smith *et al.* 2018 [206] | Ineligible study type | Conference abstract |
| Smith *et al.* 2021a [207] | Repeated dataset | Results based on secondary analysis (repeated SAGE dataset) |
| Smith *et al.* 2021b [208] | Repeated dataset | Results based on secondary analysis (repeated SAGE dataset) |
| Smith *et al.* 2022 [209] | Repeated dataset ^a^  Ineligible outcome ^b^ | Results based on secondary analysis (repeated SAGE dataset)  Does not report provide separate depression prevalence data for Ghanaian participants |
| Smith *et al.* 2022b [210] | Repeated dataset ^a^  Ineligible outcome ^b^ | Results based on secondary analysis (repeated SAGE dataset)  Targeted prevalence of suicidal ideation and suicide attempts |
| Stubbs *et al.* 2016 [211] | Ineligible outcome | Does not report the prevalence of anxiety and depressive disorders and symptoms, and/or symptoms of psychological distress |
| Stubbs *et al.* 2018 [212] | Repeated dataset ^a^  Ineligible outcome ^b^ | Results based on secondary analysis (repeated SAGE dataset)  Does not report provide separate depression prevalence data for Ghanaian participants |
| Sulemana *et al.* 2021 [213] | Ineligible assessment method | Measured symptoms of psychological distress using one question |
| Sum *et al.* 2019 [214] | Repeated dataset ^a^  Ineligible outcome ^b^ | Results based on secondary analysis (repeated SAGE dataset)  Targeted non-communicable disease dyad and triad prevalence |
| Sweetland *et al.* 2019 [215] | Ineligible outcome | Does not report separate prevalence data for adult participants aged ≥ 18 years |
| Tawiah *et al.* 2015 [216] | Ineligible condition | Targeted mental disorders and not specific mental disorders |
| Tetteh *et al.* 2020 [217] | Repeated dataset ^a^  Ineligible outcome ^b^ | Results based on secondary analysis (repeated SAGE dataset)  Targeted prevalence of visual impairment |
| Thapa *et al.* 2014 [218] | Repeated dataset | Results based on secondary analysis (repeated SAGE dataset) |
| Uzir *et al.* 2022 [219] | Ineligible outcome | Does not report the prevalence of anxiety and depressive disorders and symptoms, and/or symptoms of psychological distress |
| Vancampfort *et al.* 2018a [220] | Repeated dataset | Results based on secondary analysis (repeated SAGE dataset) |
| Vancampfort *et al.* 2018b [221] | Ineligible assessment method | Measured anxiety symptoms (exposure variable) based on one question |
| Vellakkal *et al.* 2015 [222] | Repeated dataset a  Ineligible outcome b | Results based on secondary analysis (repeated SAGE dataset)  Targeted noncommunicable disease prevalence |
| **Vousoura 2015** [223] | Ineligible study type | Dissertation |
| Waterhouse *et al.* 2016 [224] | Ineligible condition | Measured mental health |
| Wemakor *et al.* 2023a [225] | Ineligible outcome | Targeted participants aged 15-40 years, and does not report the prevalence of anxiety and depressive disorders and symptoms, and/or symptoms of psychological distress |
| Wemakor and Iddrisu 2018 [226] | Ineligible outcome | Targeted prevalence of stunting in children aged 6-23 months. Does not report the prevalence of anxiety and depressive disorders and symptoms, and/or symptoms of psychological distress |
| Wemakor and Mensah 2016 [227] | Ineligible outcome | Targeted participants aged 15-45 years, and does not report separate prevalence data for adult participants aged ≥ 18 years |
| Wemakor *et al.* 2023b [228] | Ineligible outcome | Problems with prevalence data (percentages given without numerator and denominator) |
| Winifred *et al.* 2022 [229] | Repeated dataset | Repeated dataset |
| Wombeogo 2022 [230] | Ineligible study type | Qualitative study design |
| Yeboah *et al.* 2024 [231] | Repeated dataset | Repeated dataset |
| Yirdong *et al.* 2023 [232] | Ineligible outcome | Target participants aged ≥ 15 years old but does not report separate prevalence data for adult participants aged ≥ 18 years |
| Yorke *et al.* 2023 [233] | Ineligible outcome | Does not report the prevalence of anxiety and depressive disorders and symptoms, and/or symptoms of psychological distress |
| Zagurny *et al.* 2022 [234] | Ineligible outcome | Does not report the prevalence of anxiety and depressive disorders and symptoms, and/or symptoms of psychological distress |

^a^ Primary reason for exclusion

^b^ Other reasons for exclusion

# **Appendix H. References to excluded studies**

1. **Abass K, Gyasi RM, Katey D, Frempong F and Garsonu EK** (2022) Flood exposure and psychological distress among Ghanaian adults in flood-prone settings. *Science of the Total Environment* **835**. https://doi.org/10.1016/j.scitotenv.2022.155481.
2. **Abass K, Gyasi RM, Serbeh R and Obeng B** (2023) Flood stressors and mental distress among community-dwelling adults in Ghana: A mediation model of flood-risk perceptions. *Environmental Hazards* **22**(5), 403–420. https://doi.org/10.1080/17477891.2023.2183177.
3. **Abbey EA, Mate-Kole CC, Amponsah B and Belgrave FZ** (2021) Dipo rites of passage and psychological well-being among Krobo adolescent females in Ghana: A preliminary study. *Journal of Black Psychology* **47**(6), 387–400. https://doi.org/10.1177/00957984211011307.
4. **Abbey EA and Nasidi NA** (2023) Krobo girls and dipo puberty rites of passage in the Eastern region of Ghana. *International Journal of Modern Anthropology* **2**(19), 1110–1127. https://doi.org/10.4314/ijma.v2i19.4.
5. **Abeasi DA** (2020) Depression and quality of life among family caregivers of Stroke Survivors in Ghana: The role of social support. *Archives of Mental Health* **21**(2), 83. https://doi.org/10.4103/AMH.AMH_21_19.
6. **Aberese-Ako M, Immurana M, Dalaba MA, Anumu FEY, Ofosu A and Gyapong M** (2022) The socio-economic and health effects of COVID-19 among rural and urban-slum dwellers in Ghana: A mixed methods approach. *PLoS ONE* **17**(7 July). https://doi.org/10.1371/journal.pone.0271551.
7. **Abunyewah M, Okyere SA, Opoku Mensah S, Erdiaw-Kwasie M, Gajendran T and Byrne MK** (2024) Drought impact on peri-urban farmers’ mental health in semi-arid Ghana: The moderating role of personal social capital. *Environmental Development* **49**. https://doi.org/10.1016/j.envdev.2023.100960.
8. **Achore M and Bisung E** (2022) Experiences of inequalities in access to safe water and psycho-emotional distress in Ghana. *Social Science & Medicine* **301**, 114970. https://doi.org/10.1016/j.socscimed.2022.114970.
9. **Acolatse N** (2020) Discrimination and Abuses: The impact on the mental and general health conditions of gay, lesbian, bisexual, and transgender populations in Ghana. *Texila International Journal of Academic Research* **7**(1), 30–45. https://doi.org/10.21522/TIJAR.2014.07.01.Art003.
10. **Adam L, Shani AK, Yaro PB, Adwan-Kamara L and Teg-Nefaah Tabong P** (2023) Depression and quality of life of people accused of witchcraft and living in alleged witches’ camps in Northern Ghana. *Health & Social Care in the Community* **2023**, 1–12. https://doi.org/10.1155/2023/6830762.
11. **Addai I and Andrees A Rodriguez** (2014) African political, economic. and security issues ghana social, economic and political issues. Coleen Roscoe (editor) Nova Science Publishers Chapter 3: Prevalence and Predictors of Mental Disorders in Ghana. pp. 33-60. 33–60.
12. **Adejumo OA, Iyawe IO, Akinbodewa AA, Abolarin OS and Alli EO** (2019) Burden, psychological well-being and quality of life of caregivers of end stage renal disease patients. *Ghana Med. J. (Online)* **53**(3), 190–196.
13. **Adhvaryu A, Fenske J and Nyshadham A** (2019) Early life circumstance and adult mental health. *Journal of Political Economy* **127**(4), 1516–1549. https://doi.org/10.1086/701606.
14. **Adjaye-Gbewonyo D, Rebok GW, Gallo JJ, Gross AL and Underwood CR** (2019a) Urbanicity of residence and depression among adults 50 years and older in Ghana and South Africa: An analysis of the WHO Study on Global AGEing and Adult Health (SAGE). *Aging and Mental Health* **23**(6), 660–669. https://doi.org/10.1080/13607863.2018.1450839.
15. **Adjaye-Gbewonyo D, Rebok GW, Gallo JJ, Gross AL and Underwood CR** (2020) Residence in urban and rural areas over the life course and depression among Ghanaian and South African older adults. *Health and Place* **63**. https://doi.org/10.1016/j.healthplace.2020.102349.
16. **Adjaye-Gbewonyo D, Rebok GW, Gross AL, Gallo JJ and Underwood CR** (2019b) Assessing urban-rural differences in the relationship between social capital and depression among Ghanaian and South African older adults. *PloS One* **14**(6), e0218620. https://doi.org/10.1371/journal.pone.0218620.
17. **Adjei P, Akpalu A, Laryea R, Nkromah K, Sottie C, Ohene S, and Osei A (**2015) Psychiatric features of epilepsy in Accra, Ghana. Epilepsia. 56, 100.
18. **Adjei DN, Stronks K, Adu D, Beune E, Meeks K, Smeeth L, Addo J, Owusu-Dabo E, Klipstein-Grobusch K, Mockenhaupt F, Schulze M, Danquah I, Spranger J, Bahendeka SK and Agyemang C** (2019) Cross-sectional study of association between psychosocial stressors with chronic kidney disease among migrant and non-migrant Ghanaians living in Europe and Ghana: The RODAM study. *BMJ Open* **9**(8). https://doi.org/10.1136/bmjopen-2018-027931.
19. **Adjorlolo S, Adu-Poku S, Andoh-Arthur J, Botchway I and Mlyakado BP** (2017) Demographic factors, childhood maltreatment and psychological functioning among university students’ in Ghana: A retrospective study. *International Journal of Psychology* **52**, 9–17. https://doi.org/10.1002/ijop.12248.
20. **Adzika VA, Ayim-Aboagye D and Gordh T** (2016) Pain management strategies for effective coping with Sickle Cell Disease: The perspective of patients in Ghana. *Scandinavian Journal of Pain* **12**(1), 117. https://doi.org/10.1016/j.sjpain.2016.05.005.
21. **Adzika VA, Glozah FN, Ayim-Aboagye D and Ahorlu CS** (2017) Socio-demographic characteristics and psychosocial consequences of sickle cell disease: the case of patients in a public hospital in Ghana. *Journal of Health, Population, and Nutrition* **36**(1), 4. https://doi.org/10.1186/s41043-017-0081-5.
22. **Ae-Ngibise KA, Adiibokah E, Nettey OEA, Nyame S, Doku VCK, Asante KP and Owusu-Agyei S** (2017) ‘Making the mentally ill count’, lessons from a health and demographic surveillance system for people with mental and neurological disorders in the Kintampo districts of Ghana. *International Journal of Mental Health Systems* **11**(1). https://doi.org/10.1186/s13033-017-0130-x.
23. **Agyei F, Nti F, Anago E and Avinu E** (2022) Grief and Coping Strategies of Nurses Following Patient Death at the Konongo-Odumasi Government Hospital, Ghana. *Journal of Client-Centered Nursing Care* **8**, 177–190. https://doi.org/10.32598/JCCNC.8.3.434.1.
24. **Ahorsu DK, Adjaottor ES, Yeboah FA and Opoku Y** (2021) Mental health challenges in academia: comparison between students of the various educational levels in Ghana. *Journal of Mental Health* **30**(3), 292–299. https://doi.org/10.1080/09638237.2020.1739253.
25. **Ahulu LD, Gyasi-Gyamerah AA and Anum A** (2020) Predicting risk and protective factors of generalized anxiety disorder: a comparative study among adolescents in Ghana. *International Journal of Adolescence and Youth* **25**(1), 574–584. https://doi.org/10.1080/02673843.2019.1698440.
26. **Akanko I, Abor PA and Tetteh CK** (2023) A cross-sectional study on coping strategies of frontline healthcare workers amid the COVID-19 pandemic in a developing country. *SAGE Open Medicine* **11**, 20503121231197398. https://doi.org/10.1177/20503121231197398.
27. **Alhassan A, Ziblim AR and Muntaka S** (2014) A survey on depression among infertile women in Ghana. *BMC Women’s Health* **14**(1). https://doi.org/10.1186/1472-6874-14-42.
28. **Al-Riyami AZ, Masser B, Herczenik E, Arora S, Boateng LA, Dinardo CL, Hutchinson T, Ji Y, Langi Sasongko S, Tung J-P, Panchatcharam SM and Council the IYP** (2022) Psychological impact of the COVID-19 pandemic on young professionals in blood banks and transfusion services: A global cross-sectional survey. *Vox Sanguinis* **117**(5), 685–692. https://doi.org/10.1111/vox.13236.
29. **Amankwah-Poku M, Amoah AGB, Sefa-Dedeh A and Akpalu J** (2020) Psychosocial distress, clinical variables and self-management activities associated with type 2 diabetes: a study in Ghana. *Clinical Diabetes and Endocrinology* **6**(1), 14. https://doi.org/10.1186/s40842-020-00102-7.
30. **Ambugo EA** (2014) Cross-country variation in the sociodemographic factors associated with major depressive episode in Norway, the United Kingdom, Ghana, and Kenya. *Social Science and Medicine* **113**, 154–160. https://doi.org/10.1016/j.socscimed.2014.05.022.
31. **Amegbor PM, Braimah JA, Adjaye-Gbewonyo D, Rosenberg MW and Sabel CE** (2020) Effect of cognitive and structural social capital on depression among older adults in Ghana: A multilevel cross-sectional analysis. *Archives of Gerontology and Geriatrics* **89**. https://doi.org/10.1016/j.archger.2020.104045.
32. **Amegbor PM, Kuuire VZ, Yawson AE, Rosenberg MW and Sabel CE** (2021) Social frailty and depression among older adults in Ghana: Insights from the WHO SAGE Surveys. *Research on Aging* **43**(2), 85–95. https://doi.org/10.1177/0164027520946447.
33. **Amissah CM and Nyarko K** (2020) Facing the ills of unemployment: The role of religiosity and social support. *Journal of Religion and Health* **59**(5), 2577–2594. https://doi.org/10.1007/s10943-019-00977-6.
34. **Amissah J, Amissah AB, Rockson GNY and Nakua EK** (2023) EPH225 understanding the relationship between depression and suicidal tendencies among tertiary students in Ghana. *Value in Health* **26**(6), S204. https://doi.org/10.1016/j.jval.2023.03.2571.
35. **Amissah NB, Amissah CM and Amponsah B** (2022) Assessing psychosocial distress associated with homelessness in Ghana: A springboard for interventional policy design. *Journal of Health Psychology* **27**(14), 3085–3096. https://doi.org/10.1177/13591053221082767.
36. **Amoah CA, Somhlaba NZ, Amoah B, Ansah EOA and Owusu-Ansah FE** (2022) Internet addiction and correlates among tertiary students in a sub-Saharan African Country – Case of KNUST, Ghana: A Follow Up Study. *Journal of Science and Technology (Ghana)* **40**(2), 71–86.
37. **Amoah PA, Osei-Tutu A and Adjei SB** (2023) Socio-economic and technological aspects of mental health of older persons: the role of strong and weak ties in Ghana. *Ageing & Society* **43**(11), 2650–2672. https://doi.org/10.1017/S0144686X21001859.
38. **Anim MT, Osafo J and Yirdong F** (2016) Prevalence of psychological symptoms among adults with sickle cell disease in Korle-Bu Teaching Hospital, Ghana. *BMC Psychology* **4**(1). https://doi.org/10.1186/s40359-016-0162-z.
39. **Anim-Sampong AM, Vanderpuye V, Botwe BO and Anim-Sampong S** (2021) Psychosocial impact of mastectomy on female breast cancer patients presenting at an academic radiotherapy oncology centre in Ghana. *Journal of Radiotherapy in Practice* **20**(3), 306–315. https://doi.org/10.1017/S146039692000045X.
40. **Ansah EW, Adabla M, Jerry N, Aloko EA and Hagan JE** (2023) Investigating sedentariness and health status of primary school teachers in Ghana. *BMC Health Services Research* **23**(1), 983. https://doi.org/10.1186/s12913-023-09925-3.
41. **Appiah-Poku J, Laugharne R, Mensah E, Osei Y and Burns T** (2004) Previous help sought by patients presenting to mental health services in Kumasi, Ghana. *Social Psychiatry and Psychiatric Epidemiology* **39**(3), 208–211. https://doi.org/10.1007/s00127-004-0725-9.
42. **Arku G, Luginaah I, Mkandawire P, Baiden P and Asiedu AB** (2011) Housing and health in three contrasting neighbourhoods in Accra, Ghana. *Social Science & Medicine* **72**(11), 1864–1872. https://doi.org/10.1016/j.socscimed.2011.03.023.
43. **Arokiasamy P, Uttamacharya U, Jain K, Biritwum RB, Yawson AE, Wu F, Guo Y, Maximova T, Espinoza BM, Salinas Rodríguez A, Afshar S, Pati S, Ice G, Banerjee S, Liebert MA, Snodgrass JJ, Naidoo N, Chatterji S and Kowal P** (2015) The impact of multimorbidity on adult physical and mental health in low- and middle-income countries: What does the study on global ageing and adult health (SAGE) reveal? *BMC Medicine* **13**(1). https://doi.org/10.1186/s12916-015-0402-8.
44. **Asante KO** (2012) Social support and the psychological wellbeing of people living with HIV/AIDS in Ghana. *African Journal of Psychiatry* **15**(5), 340–345. https://doi.org/10.4314/ajpsy.v15i5.42.
45. **Asante PGO, Owusu AY, Oppong JR, Amegah KE and Nketiah-Amponsah E** (2023) The psychosocial burden of women seeking treatment for breast and cervical cancers in Ghana’s major cancer hospitals. *PLOS ONE* **18**(8), e0289055. https://doi.org/10.1371/journal.pone.0289055.
46. **Ashdown-Franks G, Stubbs B, Koyanagi A, Schuch F, Firth J, Veronese N and Vancampfort D** (2019) Handgrip strength and depression among 34,129 adults aged 50 years and older in six low- and middle-income countries. *Journal of Affective Disorders* **243**, 448–454. https://doi.org/10.1016/j.jad.2018.09.036.
47. **Asiedu K, Dzasimatu SK and Kyei S** (2018) Impact of dry eye on psychosomatic symptoms and quality of life in a healthy youthful clinical sample. *Eye & Contact Lens* **44**, S404. https://doi.org/10.1097/ICL.0000000000000550.
48. **Asiedu N, Kretchy I and Asampong E** (2020) Psycho-behavioral factors associated with neurocognitive performance among people living with hiv on antiretroviral therapy in Accra, Ghana. *African Health Sciences* **20**(2), 587–596. https://doi.org/10.4314/ahs.v20i2.6.
49. **Asiwome FI** (2020, September) *Relationship between psychological distress, cognitive coping and sexual self-efficacy of cervical cancer survivors in Korle Bu Teaching Hospital, Accra, Ghana* (Thesis). University of Cape Coast. Retrieved from http://ir.ucc.edu.gh/jspui/handle/123456789/6826
50. **Asoogo C, Hoyte-Williams M, Dwobeng B, Sam D and Amankwa-Frimpong E** (2018) Experiences of patients diagnosed and living with metastatic breastcancer in Kumasi, Ghana: A lesion to learn from low-middle income country. *Journal of Global Oncology* **4**, 8s. https://doi.org/10.1200/jgo.18.40600.
51. **Atefoe E, Akotia C and Nyarko K** (2017) Religiosity, social support and social negativity as predictors of mental health of women in Ghana. *Ghana Social Science Journal* **14**.
52. **Atefoe EA and Kugbey N** (2018) Psychological functioning and well-being among Ghanaian women: A brief report. *Journal of Psychology in Africa* **28**(1), 73–76. https://doi.org/10.1080/14330237.2017.1419920.
53. **Atuoye KN and Luginaah I** (2017) Food as a social determinant of mental health among household heads in the Upper West region of Ghana. *Social Science & Medicine* **180**, 170–180. https://doi.org/10.1016/j.socscimed.2017.03.016.
54. **Awanyo L, McCarron M and Attua E** (2017) Breaking the silence: Housing and psychosocial health inequalities in three urban neighbourhoods of Accra, Ghana. *International Development Planning Review* **39**(3), 275–297. https://doi.org/10.3828/idpr.2017.8.
55. **Ayisi-Boateng NK, Blay Nguah S, Omuojine JP, Ovbiagele B and Sarfo FS** (2022) Course and characteristics of depression over a 12-month period among Ghanaians living with HIV: the EVERLAST study. *AIDS Care - Psychological and Socio-Medical Aspects of AIDS/HIV* **34**(12), 1547–1554. https://doi.org/10.1080/09540121.2022.2029821.
56. **Azure JA** (2011) Correlates of course anxiety and academic procrastination in higher education. *Global Journal of Educational Research* **10**(1), 55–65.
57. **Babagoli MA, Adu-Amankwah D, Nonterah EA, Aborigo RA, Kuwolamo I, Jones KR, Alvarez EE, Horowitz CR, Weobong B and Heller DJ** (2024) Sociodemographic and behavioral factors associated with hypertension and depression in 4 rural communities in Northern Ghana: A cross-sectional study. *Journal of Primary Care and Community Health* **15**. https://doi.org/10.1177/21501319241242965.
58. **Benneh CO, Atindanbila S, Owusu-Akyem S and Issaka EA** (2014) Gender differences in the social determinants of depression among men and women in Ghana. *International Journal of Educational Studies* **1**(2), 63–72.
59. **Bettmann JE, Prince KC, Hardy CJ and Dwumah P** (2019) Measuring Anxiety and depression in Ghanaian and U.S. college students. *Journal of Multicultural Counseling and Development* **47**(2), 119–130. https://doi.org/10.1002/jmcd.12126.
60. **Bhan N, Rao N and Raj A** (2020) Gender differences in the associations between informal caregiving and wellbeing in low-and middle-income countries. *Journal of Women’s Health* **29**(10), 1328–1338. https://doi.org/10.1089/jwh.2019.7769.
61. **Bisung E, Kangmennaang J and Luginaah I** (2018) Neighborhood structural differences and women’s mental health: An empirical study in Accra, Ghana. *Quality of Life Research* **27**(3), 661–671. https://doi.org/10.1007/s11136-017-1731-1.
62. **Błachnio A, Cudo A, Kot P, Torój M, Oppong Asante K, Enea V, Ben-Ezra M, Caci B, Dominguez-Lara SA, Kugbey N, Malik S, Servidio R, Tipandjan A and Wright MF** (2022) Cultural and psychological variables predicting academic dishonesty: A cross-sectional study in nine countries. *Ethics and Behavior* **32**(1), 44–89. https://doi.org/10.1080/10508422.2021.1910826.
63. **Blankson PK, Amanor EM, Dai-Kosi AD, Amoako E, Konadu AB, Boamah MO, Amoah GK and Parkins GE** (2021) Paediatric maxillofacial fractures in Ghana: Pattern, household cost, and distress. *International Journal of Paediatric Dentistry* **31**(5), 613–618. https://doi.org/10.1111/ipd.12734.
64. **Boadi-Kusi SB, Asamoah S, Zaabaar E, Hammond F and Ackom CK** (2023) Psychological factors associated with visual impairment. *Journal of Visual Impairment & Blindness* **117**(3), 233–245. https://doi.org/10.1177/0145482X231184435.
65. **Boima V, Tetteh J, Yorke E, Acheampong T, Mensah G, Biritwum R and Yawson AE** (2020) Older adults with hypertension have increased risk of depression compared to their younger counterparts: Evidence from the World Health Organization study of global ageing and adult health wave 2 in Ghana. *Journal of Affective Disorders* **277**, 329–336. https://doi.org/10.1016/j.jad.2020.08.033.
66. **Bonful HA and Anum A** (2019) Sociodemographic correlates of depressive symptoms: A cross-sectional analytic study among healthy urban Ghanaian women. *BMC Public Health* **19**(1). https://doi.org/10.1186/s12889-018-6322-8.
67. **Bonsu K, Kugbey N, Ayanore MA and Atefoe EA** (2019) Mediation effects of depression and anxiety on social support and quality of life among caregivers of persons with severe burns injury. *BMC Research Notes* **12**(1). https://doi.org/10.1186/s13104-019-4761-7.
68. **Boyce W, Raja S, Patranabish RG, Bekoe T, Deme-der D and Gallupe O** (2009) Occupation, poverty and mental health improvement in Ghana. *Alter* **3**(3), 233–244. https://doi.org/10.1016/j.alter.2009.03.002.
69. **Brinda EM, Rajkumar AP, Attermann J, Gerdtham UG, Enemark U and Jacob KS** (2016) Health, Social, and economic variables associated with depression among older people in low- and middle-income countries: World Health Organization study on global ageing and adult health. *American Journal of Geriatric Psychiatry* **24**(12), 1196–1208. https://doi.org/10.1016/j.jagp.2016.07.016.
70. **Cabello M, Miret M, Caballero FF, Chatterji S, Naidoo N, Kowal P, D’Este C and Ayuso-Mateos JL** (2017) The role of unhealthy lifestyles in the incidence and persistence of depression: A longitudinal general population study in four emerging countries. *Globalization and Health* **13**(1), 18. https://doi.org/10.1186/s12992-017-0237-5.
71. **Chan D, Hamamura T, Li LMW and Zhang X** (2017a) Is trusting others related to better health? an investigation of older adults across six non-western countries. *Journal of Cross-Cultural Psychology* **48**(8), 1288–1301. https://doi.org/10.1177/0022022117722632.
72. **Chan DKC, Hamamura T, Wai Li LM and Zhang X** (2017b) Effect of human development on the relationship between generalised trust and health: An international cross-sectional investigation. *The Lancet* **390**(SPEC.ISS 1), 105.
73. **Chan DKC, Zhang X, Fung HH and Hagger MS** (2015) Does emotion and its daily fluctuation correlate with depression? A cross-cultural analysis among six developing countries. *Journal of Epidemiology and Global Health* **5**(1), 65–74. https://doi.org/10.1016/j.jegh.2014.09.001.
74. **Chirwa GC, Suhrcke M and Moreno-Serra R** (2020) The impact of Ghana’s national health insurance on psychological distress. *Applied Health Economics and Health Policy* **18**(2), 249–259. https://doi.org/10.1007/s40258-019-00515-1.
75. **Clegg-Lamptey JNA, Dakubo JCB and Attobra YN** (2009) Psychosocial aspects of breast cancer treatment in Accra, Ghana. *East African Medical Journal* **86**(7), 348–353.
76. **Cole NN, Nonterah CW, Utsey SO, Hook JN, Hubbard RR, Opare-Henaku A and Fischer NL** (2015) Predictor and moderator effects of ego resilience and mindfulness on the relationship between academic stress and psychological well-being in a sample of Ghanaian college students. *Journal of Black Psychology* **41**(4), 340–357. https://doi.org/10.1177/0095798414537939.
77. **Dadzie HAN, Teye-Kwadjo E, Oppong Asante K, Amankwah-Poku M, Gyasi-Gyamerah AA, Akotia CS, Osafo J, Amankwaa-Frempong E, Roomaney R and Kagee A** (2023) Psychological factors associated with mental adjustment to breast cancer: A hospital-based observational study. *Illness, Crisis & Loss* 10541373231176018. https://doi.org/10.1177/10541373231176018.
78. **Darko G, Björkqvist K and Österman K** (2024) Workplace bullying and psychological distress in public institutions in Ghana. *European Journal of Social Science Education and Research* **11**(1), 30–50. https://doi.org/10.26417/ejser.v6i1.p62-74.
79. **de Menil V, Osei A, Douptcheva N, Hill AG, Yaro P and De-Graft Aikins A** (2012) Symptoms of common mental disorders and their correlates among women in Accra, Ghana: a population-based survey. *Ghana Medical Journal* **46**(2), 95–103.
80. **Dey N, Oti-Boadi M, Malm E, Selormey K and Owusu Ansah K** (2022) Fear of COVID-19, perceived academic stress, future anxiety, and psychological distress of Ghanaian university students: A serial mediation examination. *Journal of Psychology in Africa* **32**, 423–430. https://doi.org/10.1080/14330237.2022.2120703.
81. **Doglikuu D, Abubakari A, Yaseri M, Shakibazadeh E, Djazayery A and Mirzaei K** (2021a) Do interactions between patients’ psychological distress and adherence to dietary recommendation predict glycemic control among persons with type 2 diabetes in Ghana? *Lifestyle Medicine* **2**. https://doi.org/10.1002/lim2.22.
82. **Doglikuu D, Abubakari A, Yaseri M, Shakibazadeh E, Djazayery A and Mirzaei K** (2021b) *Does* psychological distress interact with patients’ illness and treatment perceptions to predict adherence to dietary recommendation among persons with t2dm in Ghana? A Facility Based Cross Sectional Survey. https://doi.org/10.21203/rs.3.rs-543584/v1.
83. **Donnir G and Asare-Doku W** (2021) Prevalence of psychiatric disorders and associated characteristics for recidivism: a study among prisoners in Ghana. In Australian And New Zealand Journal of Psychiatry, Vol. 55. Sage Publications LTD 1 Olivers Yard, 55 City Road, London Ec1y 1sp, England, 73–73. https://scholar.google.com/scholar?cluster=10585854959896937514&hl=en&oi=scholarr (accessed 6 May 2024)
84. **Donnir GM and Asare-Doku W** (2023) Prevalence of psychiatric disorders among sentenced prisoners in a medium security prison in Ghana: Implications for mental health assessment and service. *Criminal Behaviour and Mental Health* **33**(5), 354–368. https://doi.org/10.1002/cbm.2307.
85. **Duda RB, Anarfi JK, Adanu RMK, Seffah J, Darko R and Hill AG** (2011) The health of the ‘older women’ in Accra, Ghana: Results of the women’s health study of Accra. *Journal of Cross-Cultural Gerontology* **26**(3), 299–314. https://doi.org/10.1007/s10823-011-9148-8.
86. **Durizzo K, Asiedu E, van der Merwe A and Günther I** (2022) Economic recovery but stagnating mental health during a global pandemic? Evidence from Ghana and South Africa. *The Review of Income and Wealth* **68**(2), 563–589. https://doi.org/10.1111/roiw.12587.
87. **Dzator J** (2013) Hard times and common mental health disorders in developing countries: Insights from urban Ghana. *Journal of Behavioral Health Services and Research* **40**(1), 71–87. https://doi.org/10.1007/s11414-012-9305-z.
88. **Ed-Bansah D, Taylor A and Tagoe TA** (2023) Daytime sleepiness reflects depression, anxiety, and stress among students at the University of Ghana Medical School: *Health Sciences Investigations Journal* **4**(1), 473–480. https://doi.org/10.46829/hsijournal.2023.6.4.1.473-480.
89. **Ekem-Ferguson G, Gyimah E and Ofosuhene Mensah K** (2017) Relationship between complications of disease, depression and quality of life of type 2 diabetes patients in Ghana. *Ghana Journal of Education: Issues and Practice (GJE)* **3**, 36–53. https://doi.org/10.47963/gje.v3i.487.
90. **Ekem-Ferguson G, Swaray S, Tetteh J, Swaray A, Essuman A, Acheampong F and Yawson AE** (2022) The nexus of mental health and COVID-19-related duties among medical laboratory professionals: the mediating role of resilience. **3**, 303–311. https://doi.org/10.46829/hsijournal.2022.6.3.1.303-311.
91. **Ephraim RKD, Duah E, Nkansah C, Amoah S, Fosu E, Afrifa J, Botchway F, Okyere P, Essien-Baidoo S, Mensah K, Serwaa D, Sakyi SA, Adoba P, Fondjo LA, Ninnoni JP and Aderoju YBG** (2021) Psychological impact of covid-19 on diabetes mellitus patients in cape coast, ghana: A cross-sectional study. *Pan African Medical Journal* **40**. https://doi.org/10.11604/pamj.2021.40.76.26834.
92. **Fernandez-Nino J, Bojorquez I, Becerra-Arias C and Astudillo-Garcia C** (2019) Religious affiliation and major depressive episode in older adults: a cross-sectional study in six low- and middle- income countries. *BMC Public Health* **19**. https://doi.org/10.1186/s12889-019-6806-1.
93. **Fernandez-Nino J, Bonilla-Tinoco L, Manrique-Espinoza B, Romero-Martinez M and Sosa-Ortiz A** (2018) Work status, retirement, and depression in older adults: An analysis of six countries based on the Study on Global Ageing and Adult Health (SAGE). *SSM-Population Health* **6**, 1–8. https://doi.org/10.1016/j.ssmph.2018.07.008.
94. **Folayan MO, Abeldaño Zuniga RA, Abeldaño GF, Quadri MFA, Jafer M, Yousaf MA, Ellakany P, Nzimande N, Ara E, Al-Khanati NM, Khalid Z, Lawal F, Roque M, Lusher J, Popoola BO, Khan AT-A, Ayanore MA, Gaffar B, Virtanen JI, Aly NM, Okeibunor JC, El Tantawi M and Nguyen AL** (2022a) Is self-reported depression, HIV status, COVID-19 health risk profile and SARS-CoV-2 exposure associated with difficulty in adhering to COVID-19 prevention measures among residents in West Africa? *BMC Public Health* **22**(1), 2057. https://doi.org/10.1186/s12889-022-14429-6.
95. **Folayan MO, Abeldaño Zuñiga RA, Virtanen JI, El Tantawi M, Abeldaño GF, Ishabiyi AO, Jafer M, Al-Khanati NM, Quadri MFA, Yousaf MA, Ellakany P, Nzimande N, Ara E, Khalid Z, Lawal FB, Lusher J, Popoola BO, Idigbe I, Khan AT-A, Ayanore MA, Gaffar B, Osamika BE, Aly NM, Ndembi N and Nguyen AL** (2022b) Associations between COVID-19 testing status, non-communicable diseases and HIV status among residents of sub-Saharan Africa during the first wave of the pandemic. *BMC Infectious Diseases* **22**(1), 535. https://doi.org/10.1186/s12879-022-07498-w.
96. **Frimpong S, Bemah Antwi A, Yosia Sunindijo R, Changxin Wang C, Ampratwum G, Dansoh A, Seiwaa Boateng E, Antwi Hagan J and Annor Mensah P** (2022) Health status of young construction workers in the Global South: The case of Ghana. *Safety Science* **148**. https://doi.org/10.1016/j.ssci.2022.105673.
97. **Garin N, Koyanagi A, Chatterji S, Tyrovolas S, Olaya B, Leonardi M, Lara E, Koskinen S, Tobiasz-Adamczyk B, Ayuso-Mateos JL and Haro JM** (2016) Global multimorbidity patterns: A cross-sectional, population-based, multi-country study. *Journals of Gerontology - Series A Biological Sciences and Medical Sciences* **71**(2), 205–214. https://doi.org/10.1093/gerona/glv128.
98. **Gehlich KH, Beller J, Lange-Asschenfeldt B, Köcher W, Meinke MC and Lademann J** (2019) Fruit and vegetable consumption is associated with improved mental and cognitive health in older adults from non-Western developing countries. *Public Health Nutrition* **22**(4), 689–696. https://doi.org/10.1017/S1368980018002525.
99. **Glozah FN, Asante KO and Kugbey N** (2018) Parental involvement could mitigate the effects of physical activity and dietary habits on mental distress in Ghanaian youth. *PLoS ONE* **13**(5). https://doi.org/10.1371/journal.pone.0197551.
100. **Greif MJ and Nii-Amoo Dodoo F** (2015) How community physical, structural, and social stressors relate to mental health in the urban slums of Accra, Ghana. *Health & Place* **33**, 57–66. https://doi.org/10.1016/j.healthplace.2015.02.002.
101. **Gyasi RM** (2019) Social support, physical activity and psychological distress among community-dwelling older Ghanaians. *Archives of Gerontology and Geriatrics* **81**, 142–148. https://doi.org/10.1016/j.archger.2018.11.016.
102. **Gyasi RM, Abass K and Adu-Gyamfi S** (2020a) How do lifestyle choices affect the link between living alone and psychological distress in older age? Results from the AgeHeaPsyWel-HeaSeeB study. *BMC Public Health* **20**(1). https://doi.org/10.1186/s12889-020-08870-8.
103. **Gyasi RM, Abass K, Segbefia AY, Afriyie K, Asamoah E, Boampong MS, Adam AM and Owusu-Dabo E** (2022) A two-mediator serial mediation chain of the association between social isolation and impaired sleep in old age. *Scientific Reports* **12**(1), 22458. https://doi.org/10.1038/s41598-022-26840-5.
104. **Gyasi RM, Adam AM and Phillips DR** (2019a) Financial inclusion, health-seeking behavior, and health outcomes among older adults in Ghana. *Research on Aging* **41**(8), 794–820. https://doi.org/10.1177/0164027519846604.
105. **Gyasi RM, Hajek A, Asante F, Accam BT, Osei-Tutu S, Rahmati M, Hervie VM, Abass K and Phillips DR** (2023a) Ageing happily in Ghana: How does social inclusion contribute? *Psychogeriatrics: The Official Journal of the Japanese Psychogeriatric Society* **23**(5), 821–830. https://doi.org/10.1111/psyg.13004.
106. **Gyasi RM, Hajek A, Owusu R, James PB, Boampong MS, Accam BT, Abass K, Owusu-Dabo E and Phillips DR** (2023b) Sleep and happiness in urban-dwelling older adults in Ghana: A serial multiple mediation model of generalized anxiety and depressive symptoms. *The American Journal of Geriatric Psychiatry* **31**(11), 953–964. https://doi.org/10.1016/j.jagp.2023.05.004.
107. **Gyasi RM, Obeng B and Yeboah JY** (2020b) Impact of food insecurity with hunger on mental distress among community-dwelling older adults. *PLoS ONE* **15**(3). https://doi.org/10.1371/journal.pone.0229840.
108. **Gyasi RM, Peprah P and Appiah DO** (2020c) Association of food insecurity with psychological disorders: Results of a population-based study among older people in Ghana. *Journal of Affective Disorders* **270**, 75–82. https://doi.org/10.1016/j.jad.2020.03.088.
109. **Gyasi RM and Phillips DR** (2020) Risk of psychological distress among community-dwelling older adults experiencing spousal loss in Ghana. *The Gerontologist* **60**(3), 416–427. https://doi.org/10.1093/geront/gnz052.
110. **Gyasi RM, Yeboah AA, Mensah CM, Ouedraogo R and Addae EA** (2019b) Neighborhood, social isolation and mental health outcome among older people in Ghana. *Journal of Affective Disorders* **259**, 154–163. https://doi.org/10.1016/j.jad.2019.08.024.
111. **Hommey C, Ma J, Asamani L and Hanson P** (2020) The moderating effect of acculturation strategies on the relationship between newcomer adjustment and employee behavior. *Frontiers in Psychology* **11**. https://doi.org/10.3389/fpsyg.2020.02117.
112. **Huang K-Y, Bornheimer LA, Dankyi E and Aikins A de-Graft** (2018) Parental wellbeing, parenting and child development in Ghanaian families with young children. *Child Psychiatry and Human Development* **49**(5), 833–841. https://doi.org/10.1007/s10578-018-0799-3.
113. **Huang R, Ghose B and Tang S** (2020) Effect of financial stress on self-rereported health and quality of life among older adults in five developing countries: A cross sectional analysis of WHO-SAGE survey. *BMC Geriatrics* **20**(1). https://doi.org/10.1186/s12877-020-01687-5.
114. **Ibrahim A, Aryeetey GC, Asampong E, Dwomoh D and Nonvignon J** (2016) Erratic electricity supply (Dumsor) and anxiety disorders among university students in Ghana: A cross sectional study. *International Journal of Mental Health Systems* **10**(1). https://doi.org/10.1186/s13033-016-0053-y.
115. **Ibrahim A, Esena RK, Aikins M, O’Keefe AM and McKay MM** (2015) Assessment of mental distress among prison inmates in Ghana’s correctional system: A cross-sectional study using the Kessler Psychological Distress Scale. *International Journal of Mental Health Systems* **9**(1). https://doi.org/10.1186/s13033-015-0011-0.
116. **Jacob L, Pizzol D, Veronese N, Stubbs B and Koyanagi A** (2019) Physical injury and depression in six low- and middle-income countries: A nationally representative study. *Journal of Affective Disorders* **248**, 99–107. https://doi.org/10.1016/j.jad.2019.01.023.
117. **Jacob L, Smith L, Kostev K, Oh H, Gyasi RM, López Sánchez GF, Song T-J, Tully MA, Haro JM, Yon DK, Shin JI and Koyanagi A** (2023) Food insecurity and insomnia-related symptoms among adults from low- and middle-income countries. *Journal of Sleep Research*. https://doi.org/10.1111/jsr.13852.
118. **Judge TR, Delouize AM, Kowal P, Naidoo N, Chatterji S and Snodgrass JJ** (2021) Poor memory and depression in older adults: Results from the study on global AGEing and adult health (SAGE). *American Journal of Physical Anthropology* **174**(SUPPL 71), 52. https://doi.org/10.1002/ajpa.24262.
119. **Kaburi BB, Bio FY, Kubio C, Ameme DK, Kenu E, Sackey SO and Afari EA** (2019) Psychological working conditions and predictors of occupational stress among nurses, Salaga Government Hospital, Ghana, 2016. *The Pan African Medical Journal* **33**, 320. https://doi.org/10.11604/pamj.2019.33.320.16147.
120. **Kawada T** (2018) Post-stroke depression: Risk assessment. *Journal of the Neurological Sciences* **387**, 228. https://doi.org/10.1016/j.jns.2018.01.034.
121. **Kennedy SH, Bekele M, Berlin NL, Ranganathan K, Hamill JB, Haileselassie E, Oppong J, Newman LA and Momoh AO** (2023) A prospective evaluation of the quality of life and mental health implications of mastectomy alone on women in sub-Saharan Africa. *Annals of Surgery* **278**(5), e1080–e1086. https://doi.org/10.1097/SLA.0000000000005891.
122. **Khumalo IP, Appiah R and Wilson Fadiji A** (2022) Measuring positive mental health and depression in Africa: A variable-based and person-centred analysis of the dual-continua model. *Frontiers in Psychology* **13**. https://doi.org/10.3389/fpsyg.2022.885278.
123. **komesuor J and Meyer-Weitz A** (2023) Factors associated with mental health of internal migrants (Kayayei) in Agbogbloshie-Ghana. *BMC Women’s Health* **23**(1), 449. https://doi.org/10.1186/s12905-023-02582-y.
124. **Kploanyi EE, Dwomoh D and Dzodzomenyo M** (2020) The effect of occupational stress on depression and insomnia: A cross-sectional study among employees in a Ghanaian telecommunication company. *BMC Public Health* **20**(1). https://doi.org/10.1186/s12889-020-08744-z.
125. **Kpotosu C and Pinkrah S** (2023) COVID-19 and mental health in Ghana. https://doi.org/10.21203/rs.3.rs-3488327/v1.
126. **Krass P, Agyekum F, Boima V, Ogedegbe G and Bertelsen N** (2014) Non-compliance with medications among hypertensives in Ghana. *Annals of Global Health* **80**. https://doi.org/10.1016/j.aogh.2014.08.120.
127. **Kretchy IA** (2022) Psychological distress among healthcare professionals during the Coronavirus Disease 2019 (COVID-19) pandemic: *Health Sciences Investigations Journal* **3**(1), 283–284. https://doi.org/10.46829/hsijournal.2022.6.3.1.283-284.
128. **Kretchy IA, Boima V, Agyabeng K, Koduah A and Appiah B** (2020) Psycho-behavioural factors associated with medication adherence among male outpatients with hypertension in a Ghanaian hospital. *PLoS ONE* **15**(1). https://doi.org/10.1371/journal.pone.0227874.
129. **Kretchy IA, Osafo J, Agyemang SA, Appiah B and Nonvignon J** (2018) Psychological burden and caregiver-reported non-adherence to psychotropic medications among patients with schizophrenia. *Psychiatry Research* **259**, 289–294. https://doi.org/10.1016/j.psychres.2017.10.034.
130. **Kretchy IA, Owusu-Daaku FT, Danquah SA and Asampong E** (2015) A psychosocial perspective of medication side effects, experiences, coping approaches and implications for adherence in hypertension management. *Clinical Hypertension* **21**, 19. https://doi.org/10.1186/s40885-015-0028-3.
131. **Kugbey N, Meyer-Weitz A and Oppong Asante K** (2019) Access to health information, health literacy and health-related quality of life among women living with breast cancer: Depression and anxiety as mediators. *Patient Education and Counseling* **102**(7), 1357–1363. https://doi.org/10.1016/j.pec.2019.02.014.
132. **Kugbey N, Oppong Asante K and Meyer-Weitz A** (2020) Depression, anxiety and quality of life among women living with breast cancer in Ghana: Mediating roles of social support and religiosity. *Supportive Care in Cancer* **28**(6), 2581–2588. https://doi.org/10.1007/s00520-019-05027-1.
133. **Kunna R, San Sebastian M and Stewart Williams J** (2017) Measurement and decomposition of socioeconomic inequality in single and multimorbidity in older adults in China and Ghana: Results from the WHO study on global AGEing and adult health (SAGE). *International Journal for Equity in Health* **16**(1). https://doi.org/10.1186/s12939-017-0578-y.
134. **Kushitor MK, Peterson MB, Asante PY, Dodoo ND, Boatemaa S, Awuah RB, Agyei F, Sakyi L, Dodoo FNA and De-Graft Aikins A** (2018) Community and individual sense of trust and psychological distress among the urban poor in Accra, Ghana. *PLoS ONE* **13**(9). https://doi.org/10.1371/journal.pone.0202818.
135. **Kwawukume EY, Ghosh TS and Wilson JB** (1993) Menopausal age of Ghanaian women. *International Journal of Gynaecology and Obstetrics* **40**(2), 151–155. https://doi.org/10.1016/0020-7292(93)90376-8.
136. **Kyei K** (2017) Assessment of anxiety and depression among breast cancer patients undergoing treatment in Ghana. *Walden Dissertations and Doctoral Studies*. https://scholarworks.waldenu.edu/dissertations/4526
137. **Kyei KA, Oswald JW, Njoku AU, Kyei JB, Vanderpuye V and Tschida P** (2020) Anxiety and depression among breast cancer patients undergoing treatment in Ghana. *African Journal of Biomedical Research* **23**(2), 227–232.
138. **Lambert SD, Bowe SJ, Livingston PM, Heckel L, Cook S, Kowal P and Orellana L** (2017) Impact of informal caregiving on older adults’ physical and mental health in low-income and middle-income countries: A cross-sectional, secondary analysis based on the WHO’s Study on global AGEing and adult health (SAGE). *BMJ Open* **7**(11). https://doi.org/10.1136/bmjopen-2017-017236.
139. **Langsi R, Osuagwu UL, Goson PC, Abu EK, Mashige KP, Ekpenyong B, Ovenseri-Ogbomo GO, Chikasirimobi G T, Miner CA, Ishaya T, Oloruntoba R, Nwaeze O, Charwe DD and Agho KE** (2021) Prevalence and factors associated with mental and emotional health outcomes among Africans during the COVID-19 lockdown period-a web-based cross-sectional study. *International Journal of Environmental Research and Public Health* **18**(3), 899. https://doi.org/10.3390/ijerph18030899.
140. **Lawrence ER, Appiah-Kubi A, Lawrence HR, Lui MY, Owusu-Antwi R, Konney T and Moyer CA** (2022) “There is no joy in the family anymore”: A mixed-methods study on the experience and impact of maternal mortality on families in Ghana. *BMC Pregnancy and Childbirth* **22**(1). https://doi.org/10.1186/s12884-022-05006-1.
141. **Lestari SK** (2019, April 1) Diversity in the factors associated with ADL-related disability among older people in six middle-income countries: A Cross-Country Comparison.
142. **Li X, Guo Y, Xiao J, Liu T, Zeng W, Hu J, He G, Rong Z, Zhu Z, Wu F and Ma W** (2022) The effect of polluting cooking fuels on depression among older adults in six low- and middle-income countries. *Science of the Total Environment* **838**. https://doi.org/10.1016/j.scitotenv.2022.155690.
143. **Lin B and Okyere MA** (2020) Multidimensional energy poverty and mental health: Micro-level evidence from Ghana. *International Journal of Environmental Research and Public Health* **17**(18), 6726. https://doi.org/10.3390/ijerph17186726.
144. **Lin H, Guo Y, Kowal P, Airhihenbuwa CO, Di Q, Zheng Y, Zhao X, Vaughn MG, Howard S, Schootman M, Salinas-Rodriguez A, Yawson AE, Arokiasamy P, Manrique-Espinoza BS, Biritwum RB, Rule SP, Minicuci N, Naidoo N, Chatterji S, Qian Z, Ma W and Wu F** (2017) Exposure to air pollution and tobacco smoking and their combined effects on depression in six low- and middle-income countries. *British Journal of Psychiatry* **211**(3), 157–162. https://doi.org/10.1192/bjp.bp.117.202325.
145. **Lloyd-Sherlock P, Agrawal S, Amoakoh-Coleman M, Adom S, Adjetey-Sorsey E, Rocco I and Minicuci N** (2019) Old age and depression in Ghana: Assessing and addressing diagnosis and treatment gaps. *Global Health Action* **12**(1). https://doi.org/10.1080/16549716.2019.1678282.
146. **Lotfaliany M, Hoare E, Jacka FN, Kowal P, Berk M and Mohebbi M** (2019) Variation in the prevalence of depression and patterns of association, sociodemographic and lifestyle factors in community-dwelling older adults in six low- and middle-income countries. *Journal of Affective Disorders* **251**, 218–226. https://doi.org/10.1016/j.jad.2019.01.054.
147. **Luo M, Ding D, Bauman A, Negin J and Phongsavan P** (2020) Social engagement pattern, health behaviors and subjective well-being of older adults: an international perspective using WHO-SAGE survey data. *BMC Public Health* **20**(1), 99. https://doi.org/10.1186/s12889-019-7841-7.
148. **Majodina MZ and Johnson FA** (1983) Standardized assessment of depressive disorders (SADD) in Ghana. *The British Journal of Psychiatry* **143**, 442–446. https://doi.org/10.1192/bjp.143.5.442.
149. **Marza Florensa AM, Boateng D, Agyemang C, Levitt K and Klipstein-Grobusch K** (2019) Multi-morbidity in Ghanaian migrants in europe and residents in ghana: The RODAM study. *Transactions of the Royal Society of Tropical Medicine and Hygiene* **113**, S31–S32. https://doi.org/10.1093/trstmh/trz094.
150. **Muruthi JR, Spafford SG, Osa ML, DeLouize A, Kowal P, Biritwum R and Snodgrass JJ** (2022) Suicidal ideation in older Ghanaian women: The roles of bodily pain, functional limitation, and chronic health conditions. *International Journal of Social Psychiatry* **68**(3), 555–563. https://doi.org/10.1177/0020764021991179.
151. **Naab F** (2011) Women's representations of infertility in Ghana, University Wisconsin - Madison: 184.
152. **Naab F, Brown R and Ward EC** (2021) Culturally adapted depression intervention to manage depression among women with infertility in Ghana. *Journal of Health Psychology* **26**(7), 949–961. https://doi.org/10.1177/1359105319857175.
153. **Nkyi AK and Baaba B** (2024) Coping, health anxiety, and stress among health professionals during Covid-19, Cape Coast, Ghana. *PLoS ONE* **19**(1 January). https://doi.org/10.1371/journal.pone.0296720.
154. **Nkyi AK, Djankpa GB and Osae S** (2021) Psychological distress and life satisfaction of Konkomba widows in the Saboba District, Ghana. *Journal of Advanced Psychology* **3**(1), 1–23. https://doi.org/10.47941/japsy.526.
155. **Nonterah C, Hahn N, Utsey S, Hook J, Abrams J, Hubbard R and Opare-Henaku A** (2015) Fear of negative evaluation as a mediator of the relation between academic stress, anxiety and depression in a sample of Ghanaian college students. *Psychology & Developing Societies* **27**, 125–142. https://doi.org/10.1177/0971333614564747.
156. **Nonterah C, Hubbard R, Taasoobshirazi G, Hahn N, Peifer J and Utsey S** (2023) Collective Self-esteem and well-being among college students in Ghana. *International Perspectives in Psychology Research Practice Consultation* **13**. https://doi.org/10.1027/2157-3891/a000084.
157. **Northuis C and Ghazi L** (2020) Association between stroke prevalence and depression, functional ability, and cognitive function in six low- and middle-income countries: The who study on global ageing and adult health. *Circulation* **141**(SUPPL 1). https://doi.org/10.1161/circ.141.suppl_1.P558.
158. **Nunfam VF** (2021) Mixed methods study into social impacts of work-related heat stress on Ghanaian mining workers: A pragmatic research approach. *Heliyon* **7**(5), e06918. https://doi.org/10.1016/j.heliyon.2021.e06918.
159. **Nutakor JA, Dai B, Gavu AK and Antwi O-A** (2020) Relationship between chronic diseases and sleep duration among older adults in Ghana. *Quality of Life Research* **29**(8), 2101–2110. https://doi.org/10.1007/s11136-020-02450-4.
160. **Nutor JJ, Gyamerah AO, Alhassan RK, Duah HO, Thompson RGA, Wilson N, Harris O, Gutierrez J, Hoffmann TJ, Getahun M and Santos G-M** (2023) Influence of depression and interpersonal support on adherence to antiretroviral therapy among people living with HIV. *AIDS Research and Therapy* **20**(1), 42. https://doi.org/10.1186/s12981-023-00538-8.
161. **Nwakasi C, Brown JS, Subedi S and Darlingtina E** (2021) Depression, functional disability, and accessing health care among older Ghanaians and South Africans: A comparative study based on WHO study on global ageing and adult health (SAGE). *Aging and Mental Health* **25**(6), 1077–1085. https://doi.org/10.1080/13607863.2020.1758904.
162. **Nyarko CC, Agyarko K, Nyarko PK and Brew L** (2021) Determinants of chronic illness among aged population in Ghana: A multinomial logit approach. *Ghana Mining Journal* **21**(1), 68–75. https://doi.org/10.4314/gm.v21i1.7.
163. **Nyundo A, Manu A, Regan M, Ismail A, Chukwu A, Dessie Y, Njau T, Kaaya SF and Smith Fawzi MC** (2020) Factors associated with depressive symptoms and suicidal ideation and behaviours amongst sub-Saharan African adolescents aged 10-19 years: cross-sectional study. *Tropical Medicine and International Health* **25**(1), 54–69. https://doi.org/10.1111/tmi.13336.
164. **Obo H, Kugbey N and Atefoe E** (2021) Social support, depression, anxiety, and quality of life among persons living with type 2 diabetes: a path analysis. *SOUTH AFRICAN JOURNAL OF PSYCHOLOGY* **51**(4), 575–586. https://doi.org/10.1177/0081246320984285.
165. **Ocansey PM, Kretchy IA, Aryeetey GC, Agyabeng K and Nonvignon J** (2021) Anxiety, depression, and stress in caregivers of children and adolescents with mental disorders in Ghana and implications for medication adherence. *Ghana Medical Journal* **55**(3), 173. https://doi.org/10.4314/gmj.v55i3.1.
166. **Ofori-Atta A, Baning F, & Rosenheck R** (2014) After integrating psychotropic medications into the care of people with mental disorders in a prayer camp in Ghana, why are all the chains not falling off? South Afr. J. Psychiatry 20 (3), 105.
167. **Ojagbemi A, Akinyemi J, Wahab K, Owolabi L, Arulogun O, Akpalu J, Akpalu A, Ogbole G, Akinsanya C, Wasiu A, Tito-Ilori M, Adekunle F, Lyrea R, Akpa O, Akinyemi R, Sarfo F, Owolabi M and Ovbiagele B** (2022) Pre-stroke depression in Ghana and Nigeria: Prevalence, predictors and association with poststroke depression. *Journal of Geriatric Psychiatry and Neurology* **35**(1), 121–127. https://doi.org/10.1177/0891988720968274.
168. **Ojagbemi A, Owolabi M, Akinyemi R, Arulogun O, Akinyemi J, Akpa O, Sarfo FS, Uvere E, Saulson R, Hurst S and Ovbiagele B** (2017) Prevalence and predictors of anxiety in an African sample of recent stroke survivors. *Acta Neurological Scandinavica* **136**(6), 617–623. https://doi.org/10.1111/ane.12766.
169. **Opoku Mensah A, Asamani L and Asumeng M** (2017) The effect of marital status on psychological distress among single and partnered mothers in Ghana: The moderating role of employment status. *International Journal of Research Studies in Psychology* **6**(2). https://doi.org/10.5861/ijrsp.2017.1755.
170. **Oppong Asante K, Meyer-Weitz A and Petersen I** (2015) Correlates of psychological functioning of homeless youth in Accra, Ghana: A cross-sectional study. *International Journal of Mental Health Systems* **9**(1). https://doi.org/10.1186/1752-4458-9-1.
171. **Oppong E, Obese E, Biney R, Agbalekpor P, Hanson P, Maame Y, Aboagye M, Adakudugu E, Adanusa M, Pinkrah R, Adomah A, Osei S, Oge E, Agbenyeku M and Ameyaw E** (2021) Spectrum of anxiety and depression reported in reproductive-aged women diagnosed with gynaecological disorders at a tertiary healthcare facility in Ghana. *International Journal of Basic & Clinical Pharmacology* **10**, 1056–1061. https://doi.org/10.18203/2319-2003.ijbcp20213358.
172. **Opuni RK, Adei D, Mensah AA, Adamtey R and Agyemang-Duah W** (2023) Health needs of migrant female head porters in Ghana: Evidence from the Greater Accra and Greater Kumasi Metropolitan areas. *International Journal for Equity in Health* **22**(1), 151. https://doi.org/10.1186/s12939-023-01947-x.
173. **Osman A, Ansah-Mensah K, Amoah-Nuamah J and Atanga RA** (2023) Flood related depression and replacement of damaged household items. *Progress in Disaster Science* **18**, 100280. https://doi.org/10.1016/j.pdisas.2023.100280.
174. **Oti-Boadi M, Malm E, Dey NEY and Oppong S** (2022) Fear of COVID-19: psychological distress and coping among university students in Ghana. *Current Psychology (New Brunswick, NJ)* **41**(11), 8260–8270. https://doi.org/10.1007/s12144-021-02267-5.
175. **Owoo N and Lambon-Quayefio M** (2020) Mixed methods exploration of Ghanaian women’s domestic work, childcare and effects on their mental health. *PLoS ONE* **forthcoming**. https://doi.org/10.1371/journal.pone.0245059.
176. **Owusu-Ansah FE, Addae AA, Peasah BO, Oppong Asante K and Osafo J** (2020) Suicide among university students: prevalence, risks and protective factors. *Health Psychology and Behavioral Medicine* **8**(1), 220–233. https://doi.org/10.1080/21642850.2020.1766978.
177. **Owusu-Ansah FE, Amoah C, Addae AA, DeGraft-Adjei V, Frimpong-Manso A and Appiah-Poku J** (2023) Psychological correlates of COVID safety protocol adherence among university students. *Ghana Medical Journal* **57**(1), 49–57. https://doi.org/10.4314/gmj.v57i1.8.
178. **Owusu-Ansah FE and Nkrumah J** (2017) “Make my burden lighter”: Depression and social support in persons with disability in Ghana. *Journal of Depression and Therapy* **1**(4), 15–27. https://doi.org/10.14302/issn.2476-1710.jdt-17-1582.
179. **Panaite V and Cohen N** (2023) Does major depression differentially affect daily affect in adults from six middle-income countries: China, Ghana, India, Mexico, Russian Federation, and South Africa? *Clinical Psychological Science* 21677026231194601. https://doi.org/10.1177/21677026231194601.
180. **Peele M and Wolf S** (2020) Predictors of anxiety and depressive symptoms among teachers in Ghana: Evidence from a randomized controlled trial. *Social Science & Medicine* **253**, 112957. https://doi.org/10.1016/j.socscimed.2020.112957.
181. **Peele M and Wolf S** (2021) Depressive and anxiety symptoms in early childhood education teachers: Relations to professional well-being and absenteeism. *Early Childhood Research Quarterly* **55**, 275–283. https://doi.org/10.1016/j.ecresq.2020.11.008.
182. **Peele M, Wolf S, Behrman JR and Aber JL** (2023) Teacher depressive symptoms and children’s school readiness in Ghana. *Child Development* **94**(3), 706–720. https://doi.org/10.1111/cdev.13909.
183. **Quansah F, Frimpong JB, Sambah F, Oduro P, Anin SK, Srem-Sai M, Hagan JE and Schack T** (2022a) COVID-19 Pandemic and teachers’ classroom safety perception, anxiety and coping strategies during instructional delivery. *Healthcare* **10**(5), 920. https://doi.org/10.3390/healthcare10050920.
184. **Quansah F, Hagan JE, Ankomah F, Srem-Sai M, Frimpong JB, Sambah F and Schack T** (2022b) Relationship between covid-19 related knowledge and anxiety among university students: exploring the moderating roles of school climate and coping strategies. *Frontiers in Psychology* **13**, 820288. https://doi.org/10.3389/fpsyg.2022.820288.
185. **Quansah F, Hagan JE, Sambah F, Frimpong JB, Ankomah F, Srem-Sai M, Seibu M, Abieraba RSK and Schack T** (2022c) Perceived safety of learning environment and associated anxiety factors during covid-19 in Ghana: Evidence from physical education practical-oriented program. *European journal of investigation in health, psychology and Education* **12**(1), 28–41. https://doi.org/10.3390/ejihpe12010003.
186. **Quashie NT, D’Este C, Agrawal S, Naidoo N and Kowal P** (2019) Prevalence of angina and co-morbid conditions among older adults in six low- and middle-income countries: Evidence from SAGE Wave 1. *International Journal of Cardiology* **285**, 140–146. https://doi.org/10.1016/j.ijcard.2019.02.068.
187. **Radcliffe C, Sam A, Matos Q, Antwi S, Amissah K, Alhassan A, Ofori IP, Xu Y, Deng Y, Reynolds NR and Paintsil E** (2020) Sankofa pediatric HIV disclosure intervention did not worsen depression scores in children living with HIV and their caregivers in Ghana. *BMC Public Health* **20**(1). https://doi.org/10.1186/s12889-020-09678-2.
188. **Ramsoomar L, Gibbs A, Chirwa ED, Machisa MT, Alangea DO, Addo-Lartey AA, Dunkle K and Jewkes R** (2023) Pooled analysis of the association between mental health and violence against women: evidence from five settings in the Global South. *BMJ Open* **13**(3), e063730. https://doi.org/10.1136/bmjopen-2022-063730.
189. **Sackey J and Sanda M-A** (2009) Influence of occupational stress on the mental health of Ghanaian professional women. *International Journal of Industrial Ergonomics* **39**(5), 876–887. https://doi.org/10.1016/j.ergon.2009.04.003.
190. **Sackey J, Zhang F, Rogers B, Aryeetey R and Wanke C** (2018) Impact on depression and health‐related quality of life among people living with HIV in Accra, Ghana. *The FASEB Journal* **31**. https://doi.org/10.1096/fasebj.31.1_supplement.312.2.
191. **Saeed N and Wemakor A** (2019) Prevalence and determinants of depression in mothers of children under 5 years in Bole District, Ghana. *BMC Research Notes* **12**(1), 373. https://doi.org/10.1186/s13104-019-4399-5.
192. **Sakyi B and Johnson FA** (2022) A cross-sectional study of the psychological impact of the COVID-19 pandemic on camped refugees in Ghana. *PLoS ONE* **17**(11 November). https://doi.org/10.1371/journal.pone.0277515.
193. **Salinas-Rodríguez A, Rivera-Almaraz A, Scott A and Manrique-Espinoza B** (2020) Severity levels of disability among older adults in low-and middle-income countries: Results from the study on global ageing and adult health (SAGE). *Frontiers in Medicine* **7**. https://doi.org/10.3389/fmed.2020.562963.
194. **Sarfo FS, Agbenorku M, Adamu S, Obese V, Berchie P and Ovbiagele B** (2019) The dynamics of poststroke depression among Ghanaians. *Journal of the Neurological Sciences* **405**. https://doi.org/10.1016/j.jns.2019.07.028.
195. **Sarfo FS, Akassi J, Adamu S, Obese V and Ovbiagele B** (2017a) Burden and predictors of poststroke cognitive impairment in a sample of Ghanaian stroke Survivors. *Journal of Stroke and Cerebrovascular Diseases* **26**(11), 2553–2562. https://doi.org/10.1016/j.jstrokecerebrovasdis.2017.05.041.
196. **Sarfo FS, Jenkins C, Mensah NA, Saulson R, Sarfo-Kantanka O, Singh A, Nichols M, Qanungo S and Ovbiagele B** (2017b) Prevalence and Predictors of sleep apnea risk among Ghanaian stroke survivors. *Journal of Stroke and Cerebrovascular Diseases* **26**(7), 1602–1608. https://doi.org/10.1016/j.jstrokecerebrovasdis.2017.02.027.
197. **Sarfo FS, Jenkins C, Singh A, Owolabi M, Ojagbemi A, Adusei N, Saulson R and Ovbiagele B** (2017c) Post-stroke depression in Ghana: Characteristics and correlates. *Journal of the Neurological Sciences* **379**, 261–265. https://doi.org/10.1016/j.jns.2017.06.032.
198. **Sarfo FS, Nichols M, Qanungo S, Teklehaimanot A, Singh A, Mensah N, Saulson R, Gebregziabher M, Ezinne U, Owolabi M, Jenkins C and Ovbiagele B** (2017d) Stroke-related stigma among West Africans: Patterns and predictors. *Journal of the Neurological Sciences* **375**, 270–274. https://doi.org/10.1016/j.jns.2017.02.018.
199. **Sarfo JO and Mate-Kole C** (2014) Type 2 diabetes mellitus, depression and neuropsychological profiles among adults in Ghana. *European Journal of Medicine. Series B* **1**, 44–51. https://doi.org/10.13187/ejm.s.b.2014.1.44.
200. **Schaefer F, Blazer D, Carr K, Connor K, Burchett B, Schaefer C and Davidson J** (2007) Traumatic events and posttraumatic stress in cross-cultural mission assignments. *Journal of Traumatic Stress* **20**, 529–39. https://doi.org/10.1002/jts.20240.
201. **Selvamani Y, Sangani P and Muhammad T** (2022) Association of back pain with major depressive disorder among older adults in six low- and middle-income countries: A cross-sectional study. *Experimental Gerontology* **167**. https://doi.org/10.1016/j.exger.2022.111909.
202. **Shrum W, Mbatia PN, Yevuyibor JT, Schafer M, Walker M, Miller P and Palackal A** (2021) The burden of elders: anxiety, depression, and personal networks in two African slums. *The Journal of Nervous and Mental Disease* **209**(7), 533–536. https://doi.org/10.1097/NMD.0000000000001340.
203. **Shupler M, Baame M, Nix E, Tawiah T, Lorenzetti F, Saah J, Anderson de Cuevas R, Sang E, Puzzolo E, Mangeni J, Betang E, Twumasi M, Amenga-Etego S, Quansah R, Mbatchou B, Menya D, Asante KP and Pope D** (2022) Multiple aspects of energy poverty are associated with lower mental health-related quality of life: A modelling study in three peri-urban African communities. *SSM - Mental Health* **2**. https://doi.org/10.1016/j.ssmmh.2022.100103.
204. **Simiyu S, Bagayoko M and Gyasi RM** (2022) Associations between water, sanitation, and depression among older people in Ghana: empirical evidence from WHO-SAGE Wave 2 survey. *Aging & Mental Health* **26**(6), 1112–1119. https://doi.org/10.1080/13607863.2021.1910796.
205. **Sipsma H, Ofori-Atta A, Canavan M, Osei-Akoto I, Udry C and Bradley EH** (2013) Poor mental health in Ghana: who is at risk? *BMC Public Health* **13**(1), 288. https://doi.org/10.1186/1471-2458-13-288.
206. **Smith J, Mosley A, Relton C, Tsamenyi S, Berdie J, Chang G, Metzger A, Shannon L and Smith J** (2018) Homeopathy reduces service users’ self-reported emotional distress in a charity supported rural community clinic i*n Ghana* *Homeopathy*, Vol. 107. https://doi.org/10.1055/s-0038-1633339.
207. **Smith L, Il Shin J, McDermott D, Jacob L, Barnett Y, López-Sánchez GF, Veronese N, Yang L, Soysal P, Oh H, Grabovac I and Koyanagi A** (2021a) Association between food insecurity and depression among older adults from low- and middle-income countries. *Depression and Anxiety* **38**(4), 439–446. https://doi.org/10.1002/da.23147.
208. **Smith L, Jacob L, López-Sánchez GF, Butler L, Barnett Y, Veronese N, Soysal P, Yang L, Grabovac I, Tully MA, Shin JI and Koyanagi A** (2021b) Anxiety symptoms and mild cognitive impairment among community-dwelling older adults from low- and middle-income countries. *Journal of Affective Disorders* **291**, 57–64. https://doi.org/10.1016/j.jad.2021.04.076.
209. **Smith L, Shin JI, Butler L, Barnett Y, Oh H, Jacob L, Kostev K, Veronese N, Soysal P, Tully M, López Sánchez GF and Koyanagi A** (2022a) Physical multimorbidity and depression: A mediation analysis of influential factors among 34,129 adults aged ≥50 years from low- and middle-income countries. *Depression and Anxiety* **39**(5), 376–386. https://doi.org/10.1002/da.23250.
210. **Smith L, Shin JI, Carmichael C, Jacob L, Kostev K, Grabovac I, Barnett Y, Butler L, Lindsay RK, Pizzol D, Veronese N, Soysal P and Koyanagi A** (2022b) Association of food insecurity with suicidal ideation and suicide attempts in adults aged ≥50 years from low- and middle-income countries. *Journal of Affective Disorders* **309**, 446–452. https://doi.org/10.1016/j.jad.2022.04.109.
211. **Stubbs B, Koyanagi A, Schuch FB, Firth J, Rosenbaum S, Veronese N, Solmi M, Mugisha J and Vancampfort D** (2016) Physical activity and depression: a large cross-sectional, population-based study across 36 low- and middle-income countries. *Acta Psychiatrica Scandinavica* **134**(6), 546–556. https://doi.org/10.1111/acps.12654.
212. **Stubbs B, Vancampfort D, Firth J, Schuch FB, Hallgren M, Smith L, Gardner B, Kahl KG, Veronese N, Solmi M, Carvalho AF and Koyanagi A** (2018) Relationship between sedentary behavior and depression: A mediation analysis of influential factors across the lifespan among 42,469 people in low- and middle-income countries. *Journal of Affective Disorders* **229**, 231–238. https://doi.org/10.1016/j.jad.2017.12.104.
213. **Sulemana I, Doabil L and Anarfo EB** (2021) Psychological distress in Ghana: Are unemployed people more afflicted? *Journal of Health Psychology* **26**(10), 1587–1596. https://doi.org/10.1177/1359105319883911.
214. **Sum G, Salisbury C, Koh GC-H, Atun R, Oldenburg B, Mcpake B, Vellakkal S and Lee JT** (2019) Implications of multimorbidity patterns on health care utilisation and quality of life in middleincome countries: Cross-sectional analysis. *Journal of Global Health* **9**(2). https://doi.org/10.7189/jogh.09.020413.
215. **Sweetland AC, Norcini Pala A, Mootz J, Kao JC-W, Carlson C, Oquendo MA, Cheng B, Belkin G and Wainberg M** (2019) Food insecurity, mental distress and suicidal ideation in rural Africa: Evidence from Nigeria, Uganda and Ghana. *International Journal of Social Psychiatry* **65**(1), 20–27. https://doi.org/10.1177/0020764018814274.
216. **Tawiah PE, Adongo PB and Aikins M** (2015) Mental Health-related stigma and discrimination in ghana: experience of patients and their caregivers. *Ghana Medical Journal* **49**(1), 30–36. https://doi.org/10.4314/gmj.v49i1.6.
217. **Tetteh J, Fordjour G, Ekem-Ferguson G, Yawson AO, Boima V, Entsuah-Mensah K, Biritwum R, Essuman A, Mensah G and Yawson AE** (2020) Visual impairment and social isolation, depression and life satisfaction among older adults in Ghana: Analysis of the WHO’s Study on global AGEing and adult health (SAGE) Wave 2. *BMJ Open Ophthalmology* **5**(1). https://doi.org/10.1136/bmjophth-2020-000492.
218. **Thapa SB, Martinez P and Clausen T** (2014) Depression and its correlates in South Africa and Ghana among people aged 50 and above: Findings from the WHO study on global ageing and adult health. *African Journal of Psychiatry (South Africa)* **17**(6). https://doi.org/10.4172/Psychiatry.1000167.
219. **Uzir MUH, Bukari Z, Jerin I, Hasan N, Abdul Hamid AB and Ramayah T** (2022) Impact of COVID-19 on psychological distress among SME owners in Ghana: Partial least square–structural equation modeling (PLS-SEM) approach. *Journal of Community Psychology* **50**(3), 1282–1314. https://doi.org/10.1002/jcop.22716.
220. **Vancampfort D, Stubbs B, Herring MP, Hallgren M and Koyanagi A** (2018a) Sedentary behavior and anxiety: Association and influential factors among 42,469 community-dwelling adults in six low- and middle-income countries. *General Hospital Psychiatry* **50**, 26–32. https://doi.org/10.1016/j.genhosppsych.2017.09.006.
221. **Vancampfort D, Stubbs B, Mugisha J, Firth J, Schuch FB and Koyanagi A** (2018b) Correlates of sedentary behavior in 2,375 people with depression from 6 low- and middle-income countries. *Journal of Affective Disorders* **234**, 97–104. https://doi.org/10.1016/j.jad.2018.02.088.
222. **Vellakkal S, Millett C, Basu S, Khan Z, Aitsi-Selmi A, Stuckler D and Ebrahim S** (2015) Are estimates of socioeconomic inequalities in chronic disease artefactually narrowed by self-reported measures of prevalence in low-income and middle-income countries? Findings from the WHO-SAGE survey. *Journal of Epidemiology and Community Health* **69**(3), 218–225. https://doi.org/10.1136/jech-2014-204621.
223. **Vousoura E.** (2015) Psychological distress among mothers of young children in rural Ghana and Uganda and its association with child health and nutritional status, ProQuest Inf. Learn. 76.
224. **Waterhouse P, Hill AG and Hinde A** (2016) Childbearing and economic work: The health balance of women in Accra, Ghana. *Maternal and Child Health Journal* **20**(2), 408–421. https://doi.org/10.1007/s10995-015-1839-2.
225. **Wemakor A, Bukari M and Atariba R** (2023a) Household food insecurity, low maternal social support and maternal common mental disorders in East Mamprusi Municipality, Ghana. *BMC Public Health* **23**(1), 1255. https://doi.org/10.1186/s12889-023-16157-x.
226. **Wemakor A and Iddrisu H** (2018) Maternal depression does not affect complementary feeding indicators or stunting status of young children (6-23 months) in Northern Ghana. *BMC Research Notes* **11**(1), 408. https://doi.org/10.1186/s13104-018-3528-x.
227. **Wemakor A and Mensah KA** (2016) Association between maternal depression and child stunting in Northern Ghana: A cross-sectional study. *BMC Public Health* **16**(1). https://doi.org/10.1186/s12889-016-3558-z.
228. **Wemakor A, Mohammed IA and Awuni V** (2023b) Determinants of household food insecurity and depression in mothers: Evidence from Ghana. *Advances in Public Health* **2023**, 1–9. https://doi.org/10.1155/2023/6691810.
229. **Winifred A-D, Jane RL, Brian K, Amponsah-Tawiah K and Carole J** (2022) Mental health and workplace factors: comparison of the Ghanaian and Australian mining industry. *BMC Health Services Research* **22**(1), 322. https://doi.org/10.1186/s12913-022-07712-0.
230. **Wombeogo M** (2022) Parental anxiety of perceived disorder and application of concoctions on infant pulsating anterior fontanelle in the Tamale Metropolis, Ghana. *Texila International Journal of Nursing* **8**(1), 18–11. https://doi.org/10.21522/TIJNR.2015.08.01.Art003.
231. **Yeboah K, Gyamfi T and Agyekum JA** (2024) Severe depression is associated with decreased levels of serum brain-derived neurotrophic factor in type 2 diabetes patients in Ghana. *Alexandria Journal of Medicine* **60**(1), 17–25. https://doi.org/10.1080/20905068.2024.2314796.
232. **Yirdong F, Anim MT, Nkyi AK and Ocansey F** (2023) Religious coping and depressive symptoms in people living with HIV in Ghana. *Mental Health, Religion & Culture* **26**(1), 34–48. https://doi.org/10.1080/13674676.2022.2164567.
233. **Yorke E, Boima V, Ganu V, Tetteh J, Twumasi L, Ekem-Ferguson G, Kretchy I and Mate-Kole CC** (2023) The mediating role of quality of life on depression and medication adherence among patients with type 2 diabetes mellitus: A cross-sectional study. *Health Science Reports* **6**(9), e1539. https://doi.org/10.1002/hsr2.1539.
234. **Zagurny ESF, Compton SD, Dzomeku V, Cannon LM, Omolo T and Munro-Kramer ML** (2022) Understanding stalking among university students in Ghana: A mixed-methods study. *Journal of Interpersonal Violence* **37**(15–16), NP13045–NP13066. https://doi.org/10.1177/08862605211001485.

# **Appendix I. Funnel plot for publication bias for anxiety disorders and symptoms**


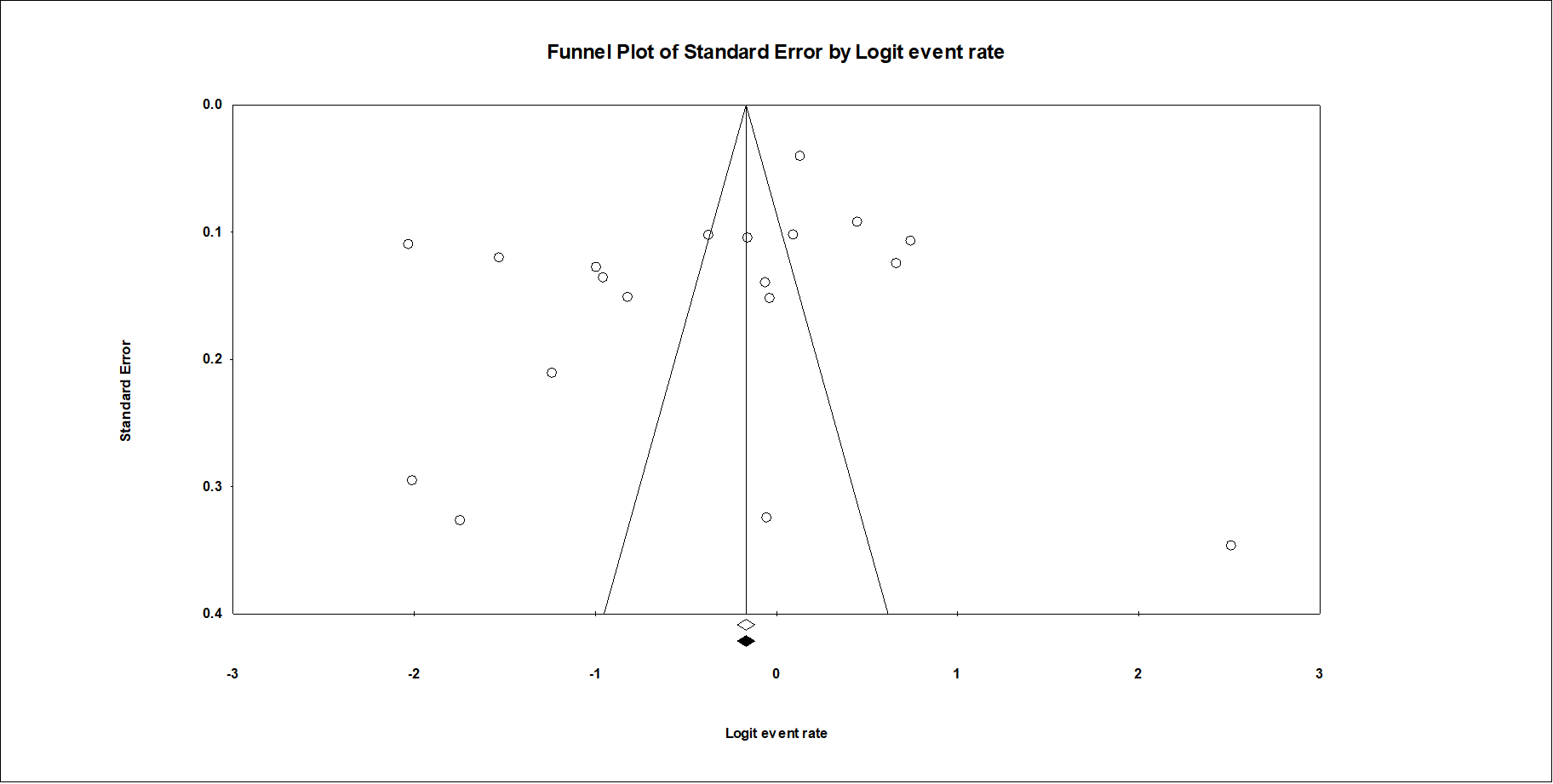


# **Appendix J. Funnel plot for publication bias for depressive disorders and symptoms**


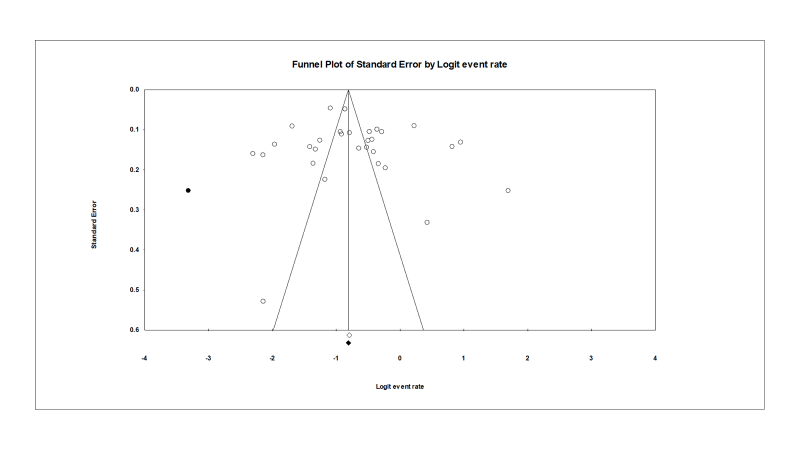


# **Appendix K. Forest and funnel plot for publication bias for symptoms of psychological distress**


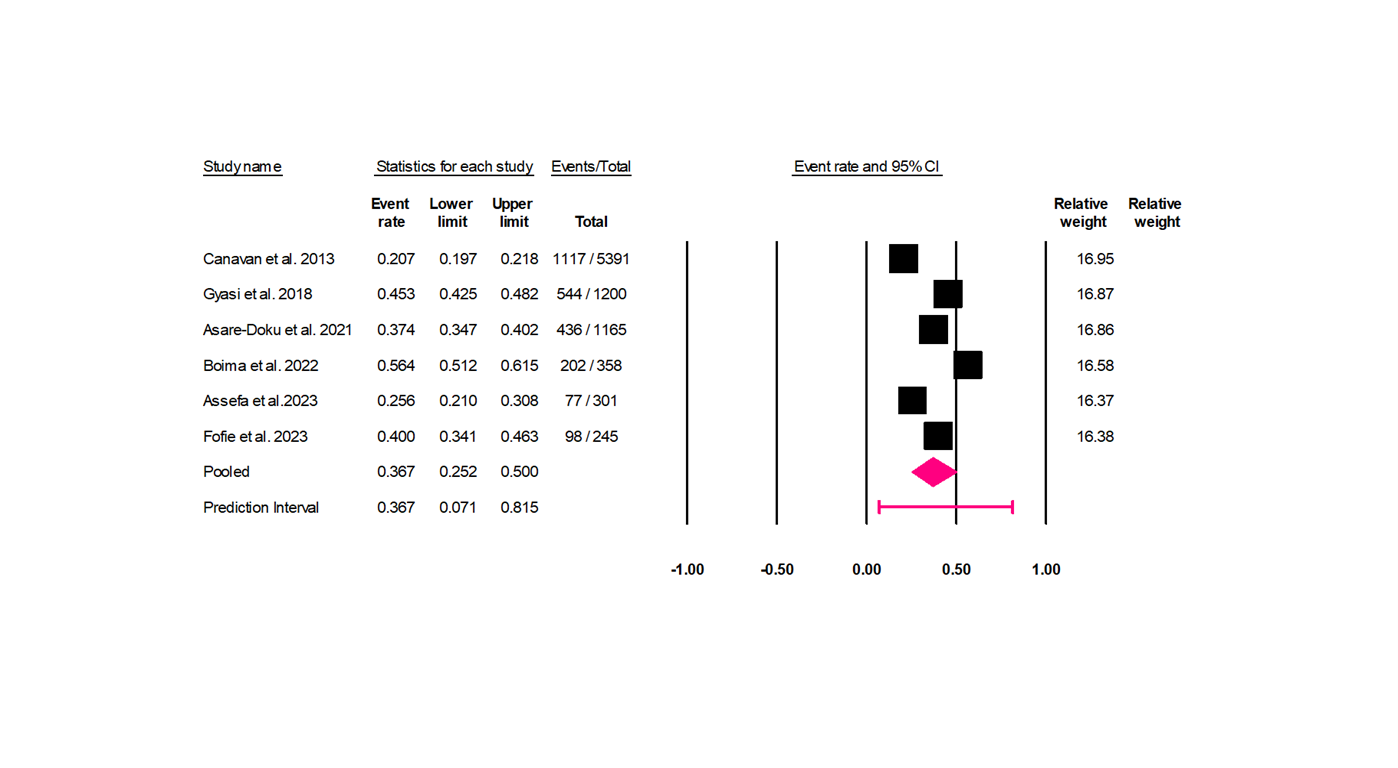


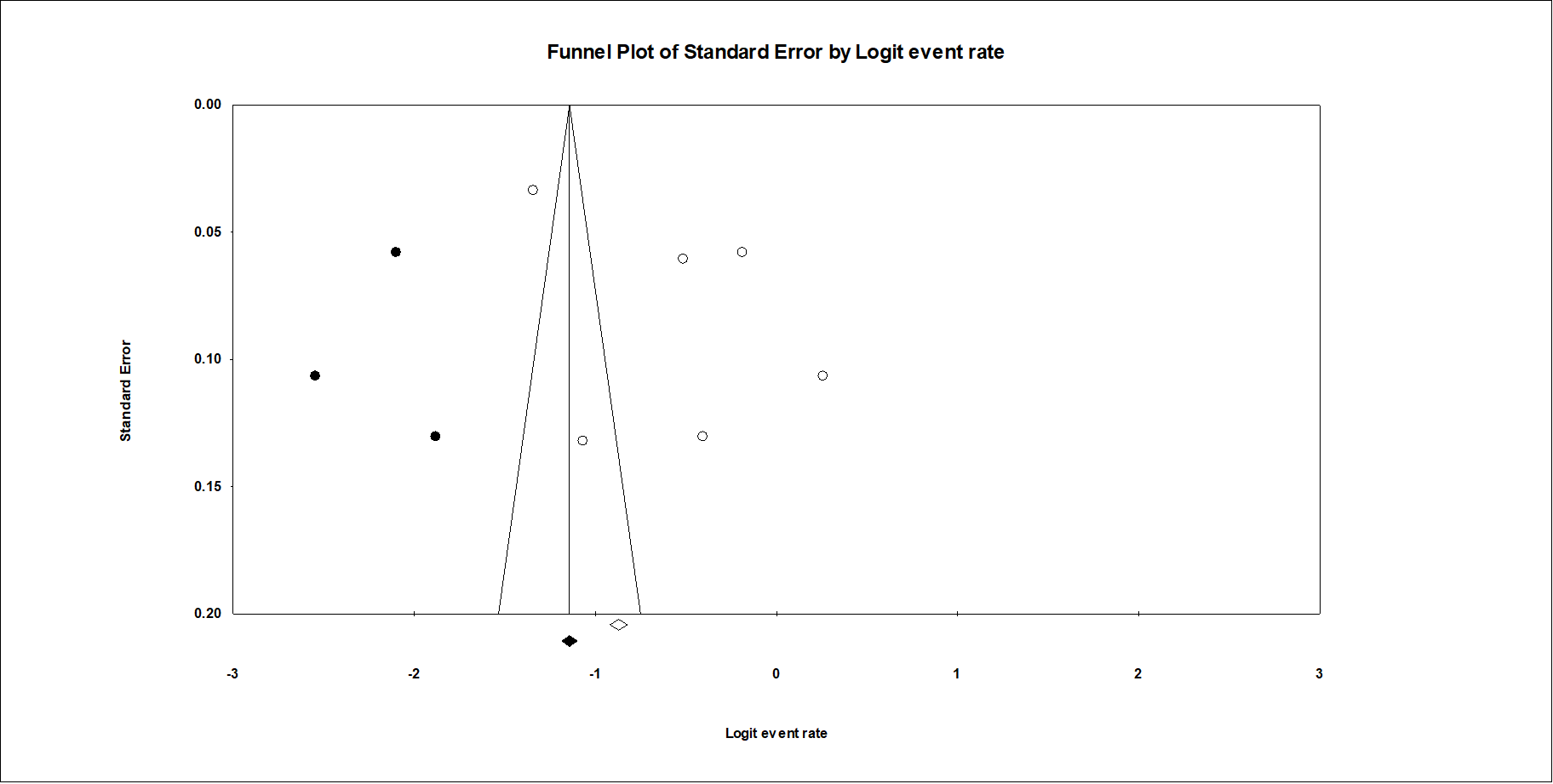


# **Appendix L. Subgroup analyses of prevalence of anxiety disorders and symptoms (n=19)**

| **Subgroup** | **Categories** | **No. of studies** | **Prevalence (95% CI)** | **I^2^ (%)** | **P values within subgroups** | **Q (p values across subgroups** |
| --- | --- | --- | --- | --- | --- | --- |
| Chronic physical conditions | Yes | 6 | 59.0% (42.3-73.8%) | 97.0 | <0.001 | **7.118 (0.008)** |
|  | No | 13 | 32.2% (23.1-42.9%) | 98.2 | <0.001 |  |
| Type of chronic physical conditions | Breast cancer | 2 | 75.9% (47.8-91.5%) | 97.9 | <0.001 | **9.926 (0.019)** |
|  | HIV/AIDS | 3 | 43.9% (22.8-64.7%) | 97.0 | <0.001 |  |
|  | Hypertension | 1 | 67.8% (28.1-91.9%) | 0.00 | 1.000 |  |
|  | None | 13 | 32.2% (22.8-43.3%) | 98.2 | <0.001 |  |
| Method of mental health assessment | Self-report screening tool | 18 | 40.9 (32.0-50.3%) | 98.0 | <0.001 | 0.285 (0.594) |
|  | Diagnostic interview | 1 | 30.6 (8.1-68.8%) | 0.00 | 1.000 |  |
| Sample size | < 384 | 12 | 39.8 (28.8-51.9%) | 96.0 | <0.001 | 0.020 (0.887) |
|  | ≥ 384 | 7 | 41.2 (27.3-56.6%) | 99.0 | <0.001 |  |
| Study design | Cross-sectional | 18 | 41.9 (32.2-51.2%) | 98.0 | <0.001 | 2.602 (0.107) |
|  | Case-control | 1 | 14.9 (3.1-48.5%) | 0.00 | 1.000 |  |
| Quality assessment | Low | 2 | 27.3 (10.3-55.2%) | 97.2 | <0.001 | 1.880 (0.391) |
|  | Moderate | 11 | 38.6 (27.6-50.9) | 98.3 | <0.001 |  |
|  | High | 6 | 48.1 (32.2-64.5%) | 96.2 | <0.001 |  |
| Time period of data collection | <2020 | 6 | 55.8 (42.6-68.2%) | 97.0 | <0.001 | **16.568 (0.000)** |
|  | ≥ 2020 | 9 | 26.1 (18.7-35.2%) | 96.5 | <0.001 |  |
|  | Not reported | 4 | 52.8 (37.4-67.8%) | 78.7 | 0.003 |  |
| Region (s) | Ashanti | 4 | 38.8% (26.3-53.1%) | 97.1 | <0.001 | **39.553 (0.000)** |
|  | Ashanti and Central | 1 | 14.9% (4.6-38.9%) | 0.00 | 1.000 |  |
|  | Central | 2 | 35.6% (19.7-55.5%) | 83.2 | <0.001 |  |
|  | Greater Accra | 5 | 60.2% (46.9-72.2%) | 92.1 | <0.001 |  |
|  | Greater Accra and Ashanti | 1 | 67.8 (40.1-86.8%) | 0.00 | 1.000 |  |
|  | Greater Accra and Central | 1 | 27.0 (10.5-53.9%) | 0.00 | 1.000 |  |
|  | Northern and Eastern | 1 | 66.0 (38.1-86.0%) | 0.00 | 1.000 |  |
|  | Oti and Volta | 1 | 53.3 (27.0-77.9%) | 0.00 | 1.000 |  |
|  | Multiple locations | 3 | 13.6% (7.4-23.8%) | 79.9 | 0.007 |  |
| Population type | General population | 2 | 28.0% (9.7-58.4%) | 99.7 | <0.001 | 3.022 (0.221) |
|  | Special population | 16 | 43.9% (33.1-55.4%) | 97.1 | <0.001 |  |
|  | Both General and special population | 1 | 14.9% (2.5%-54.3%) | 0.00 | 1.000 |  |

Bold data indicate statistically significant

# **Appendix M. Regional distribution of prevalence of anxiety disorders and symptoms**


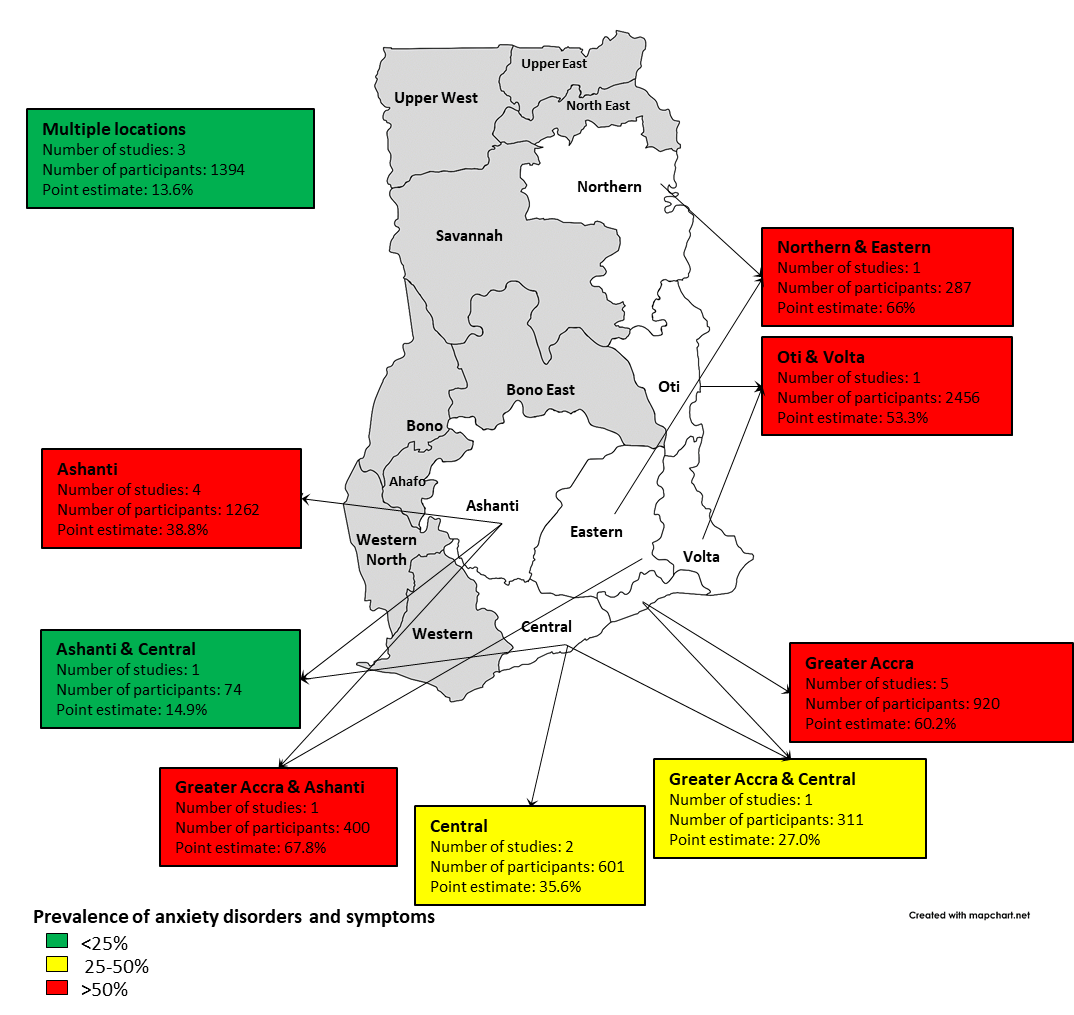


# **Appendix N. Subgroup analyses of prevalence of depressive disorders and symptoms (n=30)**

| **Subgroup** | **Categories** | **No. of studies** | **Prevalence (95% CI)** | **I^2^ (%)** | **P values within subgroups** | **Q (p values across subgroups** |
| --- | --- | --- | --- | --- | --- | --- |
| Chronic physical conditions | Yes | 10 | 37.4% (27.8-48.1%) | 96.9 | <0.001 | 1.542 (0.463) |
|  | No | 19 | 31.4% (25.0-38.7%) | 97.5 | <0.001 |  |
|  | Both^^[[1]](#footnote-1)^^ | 1 | 22.2% (6.7-53.0%) | 0.00 | 1.000 |  |
| Type of chronic physical conditions | Breast cancer | 2 | 63.3% (38.6-82.5%) | 98.3 | <0.001 | 8.293 (0.217) |
|  | Kidney disease | 1 | 44.3% (16.1-76.7%) | 0.00 | 1.000 |  |
|  | HIV/AIDS | 4 | 34.0% (20.4-50.8%) | 97.0 | <0.001 |  |
|  | Hypertension | 2 | 22.3% (9.6-43.8%) | 98.1 | <0.001 |  |
|  | Type-2-diabetes | 1 | 31.3% (10.2-64.5%) | 0.00 | 1.000 |  |
|  | Both | 1 | 22.2% (6.6-53.5%) | 0.00 | 1.000 |  |
|  | None | 19 | 31.4% (24.9-38.8%) | 97.5 | <0.001 |  |
| Method of mental health assessment | Self-report screening tool | 29 | 33.0% (27.5-38.9%) | 97.4 | <0.001 | 0.009 (0.924) |
|  | Diagnostic interview | 1 | 34.5% (11.7-67.6%) | 0.00 | 1.000 |  |
| Sample size | < 384 | 18 | 39.4% (32.9-47.9%) | 96.2 | <0.001 | **7.219 (0.007)** |
|  | ≥ 384 | 12 | 25.0% (18.7-32.6%) | 97.7 | <0.001 |  |
| Study design | Case-control | 2 | 16.7% (6.4-37.2%) | 62.5 | 0.103 | 2.963 (0.227) |
|  | Cross-sectional | 27 | 34.4% (28.6-40.8%) | 97.5 | <0.001 |  |
|  | Cross-sectional baseline | 1 | 28.3% (9.0-61.1%) | 0.00 | 1.000 |  |
| Quality Assessment | Low | 3 | 34.0% (18.6-53.6%) | 78.5 | 0.010 | 1.243 (0.537) |
|  | Moderate | 18 | 35.3% (28.2-43.2%) | 97.7 | <0.001 |  |
|  | High | 9 | 28.4% (19.9-38.6%) | 96.3 | <0.001 |  |
| Time period of data collection | <2020 | 10 | 36.9% (28.1-46.6%) | 97.8 | <0.001 | **12.678(0.005)** |
|  | ≥ 2020 | 10 | 22.6% (16.4-30.2%) | 95.7 | <0.001 |  |
|  | <2020-2020 | 1 | 22.2% (7.7-49.6%) | 0.00 | 1.000 |  |
|  | Not reported | 9 | 43.6% (33.8-53.9%) | 93.5 | <0.001 |  |
| Region | Ashanti | 5 | 37.4% (26.3%-50.0%) | 97.3 | <0.001 | **39.912 (0.000)** |
|  | Ashanti and Central | 1 | 10.5% (2.5%-35.3%) | 0.00 | 0.140 |  |
|  | Bono | 1 | 22.2% (8.2-47.7%) | 0.00 | 1.000 |  |
|  | Central | 2 | 31.4% (16.8-5.0%) | 54.2 | <0.001 |  |
|  | Greater Accra | 9 | 46.1% (36.5%-56.0%) | 91.2 | <0.001 |  |
|  | Greater Accra and Ashanti | 2 | 17.9% (8.7-33.1%) | 97.4 | <0.001 |  |
|  | Greater Accra and Central | 1 | 19.6% (7.1-43.9%) | 00.0 | <0.001 |  |
|  | Northern and Eastern | 1 | 72.1% (44.8-89.2%) | 0.00 | 1.000 |  |
|  | Oti and Volta | 1 | 25.2% (9.8-51.2%) | 0.00 | 1.000 |  |
|  | Volta | 3 | 41.7% (26.7-58.5%) | 97.9 | <0.001 |  |
|  | Ahafo, Upper East, and Volta | 1 | 15.6% (5.6-36.8%) | 0.00 | 1.000 |  |
|  | Multiple locations | 3 | 13.9% (7.5-24.2%) | 88.1 | <0.001 |  |
| Population type | General population | 4 | 19.9% (11.5-32.1%) | 97.2 | <0.001 | **9.426 (0.009)** |
|  | Specific population | 24 | 37.7% (31.6-44.2%) | 96.9 | <0.001 |  |
|  | Both general and special population | 2 | 16.9% (6.8-36.1%) | 62.5 | 0.103 |  |

Bold data indicate statistically significant.

**Appendix O. Regional distribution of prevalence of depressive disorders and symptoms**


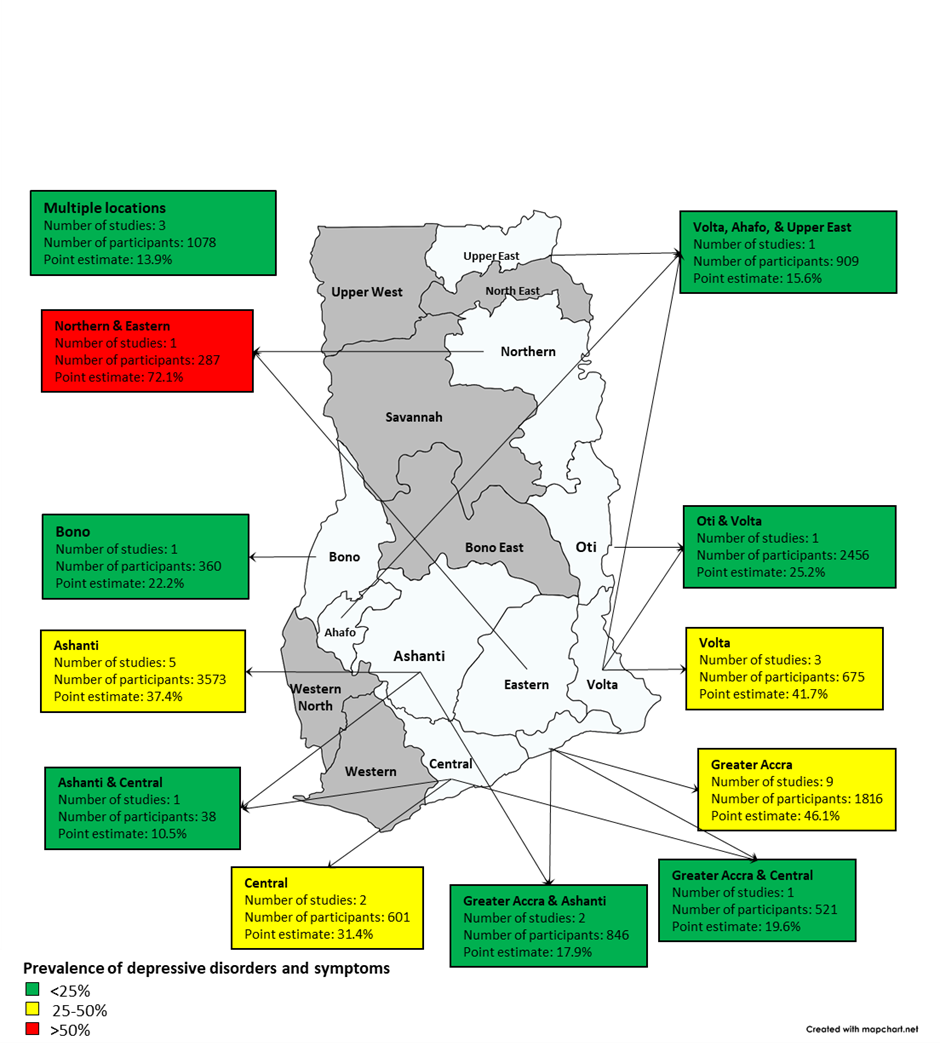


1. Both: Type-2 diabetes and non-diabetic controls [↑](#footnote-ref-1)
